# Wetting Properties of Rhamnolipid and Surfactin Mixtures with Triton X-165

**DOI:** 10.3390/molecules27154706

**Published:** 2022-07-23

**Authors:** Edyta Rekiel, Anna Zdziennicka, Katarzyna Szymczyk, Bronisław Jańczuk

**Affiliations:** Department of Interfacial Phenomena, Faculty of Chemistry, Institute of Chemical Sciences, Maria Curie-Skłodowska University in Lublin, Maria Curie-Skłodowska Sq. 3, 20-031 Lublin, Poland; edyta.rekiel@poczta.umcs.lublin.pl (E.R.); anna.zdziennicka@mail.umcs.pl (A.Z.); katarzyna.szymczyk@mail.umcs.pl (K.S.)

**Keywords:** contact angle, biosurfactants, nonionic surfactant, adhesion work, adsorption Gibbs free energy

## Abstract

The wetting properties of the rhamnolipid and surfactin mixtures with Triton X-165 were considered based on the contact angle measurements of their aqueous solution on the polytetrafluoroethylene (PTFE), polymethyl methacrylate (PMMA) and quartz (Q) surfaces. The obtained contact angle isotherms were described by the exponential function of the second order as well as by Szyszkowski equation in some cases. Using the contact angle isotherms of individual biosurfactants and TX165 as well as the earlier obtained isotherms of their surface tension the contact angle isotherms of the biosurfactants mixtures with TX165 were deduced. As follows the presence of the maxima on the contact angle isotherms of the biosurfactants mixtures with TX165 is justified. They do not prove negative adsorption of the biosurfactant and TX165 at the interfaces. However, the mutual exchange of the biosurfactant and TX165 molecules is observed in the layers at the interfaces. The concentration of the studied mixtures at the PTFE-solution interface was established to be close to that at the solution-air one but that at the PTFE-air is equal to zero. However, the concentration of the studied mixtures at the PMMA-solution and quartz-solution is greater than zero. The concentration at the PMMA(quartz)-air and PMMA(quartz)-solution interfaces is smaller than that at the solution-air one.

## 1. Introduction

The demand for surfactants of useful activity in various fields is constantly growing. Unfortunately, many types of synthetic surfactants, besides their excellent wetting, emulsifying and foaming properties, so useful in their practical application, are dangerous for the natural environment [1]. This is due to the toxic properties of many synthetic surfactants and the difficulty of their degradation. For this reason, biosurfactants, which are mainly produced by bacteria or fungi, are of increasing interest. Biosurfactants are characterized by numerous specific properties, such as great resistance to the temperature, pH and electrolyte concentration changes [2,3]. They exhibit also great adsorption activity at the interfaces and aggregation activity in the water environment [4,5]. Apart from these properties, biosurfactants are characterized by small toxicity and large biodegradability [6]. For this reason, biosurfactants are increasingly used in the petroleum, pharmaceutical, medical and food industries [7,8,9,10,11,12]. Of the biosurfactants rhamnolipid (RL) and surfactin (SF) are the most widely applied [13,14,15,16]. Unfortunately, the relatively high cost of obtaining biosurfactants is an obstacle in their application. In order to reduce the cost of biosurfactants application in practice, their mixtures with non-toxic synthetic surfactants can be used [17,18,19]. The surfactants mixtures application is correlated, among others to their adsorption, aggregation and wetting properties. However, particularly their wetting properties are least known.

According to the Young’s theory [20] the wettability of the solids by different liquids depends on the liquid-air, solid-air and solid-liquid interface tension. In the case of aqueous solution of the surfactants mixture the wettability of solids changes as a function of surfactants mixture concentration due to the adsorption of surfactants at the mentioned interfaces [21,22]. As a result of adsorption, the water-air and solid-water interface tensions change and the solid-air interface tension can also change [23]. These changes depend on the magnitude of the adsorption, the orientation of surfactant molecules in the interface layers, their packing and the composition of the adsorption mixed layers [24].

In the literature it is difficult to find the data about the adsorption properties of the surface layer at the solid-air and solid-water interfaces. In our previous studies [24] it was found out, among others, that in the case of the RL and SF mixtures with Triton X-165 (TX165) the concentration and composition of the mixed monolayer at the water-air interface can be predicted based on the isotherms of the surface tension of the aqueous solution of a particular component of the biosurfactants and TX165 mixtures as well as the tendency of this mixture towards adsorption at the water-air interface.

As mentioned above the wettability of the solids depends not only on the water-air interface tension but also the solid-air and solid-water ones. The contact angle of the liquid on the solids surface is a measure of the wetting process. Thus, the purpose of our studies was to measure the contact angle in the solid-solution drop-air system for the aqueous solution of the biosurfactant with the TX165 mixtures, the possibility to predict the contact angle for the solution mixtures based on the isotherms of the contact angle of individual mixture components as well as the adsorption amount at the solid-air and solid-water interfaces. For these studies polytetrafluoroethylene (PTFE), polymethyl methacrylate (PMMA) and quartz (Q) were chosen. These solids were treated as models for the studies of wetting properties of the biosurfactants mixtures with TX165. Of them PTFE is a low energetic apolar solid, PMMA monopolar and quartz bipolar one. It should be emphasized that PTFE and PMMA are used in medicine as implants [25] and they are also treated as substitutes in human skin wettability [26]. The contact angle isotherms obtained for the studied mixtures were described by the exponential function of the second order and the Szyszkowski equation modified by us. The obtained isotherms were also considered with regard to the contribution of individual mixture components to the water surface tension reduction as well as in the contact angle changes. This contribution was also applied for determination of the adsorption layer composition at the solid-air and solid-water interfaces. The amount of adsorption was calculated using the Gibbs isotherm equation. The isotherms of the Gibbs surface excess concentration were used for the determination of the Gibbs standard free energy of adsorption at the solid-air and solid-water interfaces. This energy was also calculated based on the constant in the Szyszkowski equation.

## 2. Results and Discussion

### 2.1. Adsorption and Wetting Properties of TX165, RL and SF

In order to understand the wetting properties of TX165 + RL and TX165 + SF mixtures, it is necessary to determine the influence of individual components of the mixture on the value of the contact angle of their aqueous solutions for different types of solids.

According to van Oss et al. [27,28], solids can be divided into three groups, namely apolar, monopolar and bipolar. This division is related to the surface tension of solids ($\gamma_{SV}$). Van Oss et al. proposed that the surface tension of solids and liquids can be treated as the sum of the Lifshitz-van der Waals ($\gamma^{LW}$) and acid-base ($\gamma^{AB}$) components. The $\gamma^{AB}$ component was assumed by van Oss et al. [27,28] as equal to the geometric mean of the electron-acceptor ($\gamma^{+}$) and electron-donor ($\gamma^{-}$) parameters. In turn, in the case of the surfactants van Oss and Constanzo [29] assumed that the surfactants surface tension depends on the orientation of their molecules toward the air phase. If the surfactant molecules are oriented toward the air phase by their hydrophobic part, then the surface tension of the surfactant is called the surfactant tail surface tension ($\gamma_{T}$). In the case of the surfactant molecules orientation toward the air phase by the hydrophilic group the surface tension of surfactants can be expressed as the surfactant head surface tension ($\gamma_{H}$). Taking into account the van Oss et al. approach [27,28] to the surface tension of liquids and solids, particular types of solids can be defined. The surface tension of apolar solids results from only the Lifshitz-van der Waals intermolecular interactions. As the contribution of the dipole and induced dipole interactions to the surface tension is smaller than 2% [27,28,29], according to Fowkes [30] the surface tension of apolar solids results practically from the London forces. The apolar solids include PTFE. The surface tension of the monopolar solids such as PMMA also results from only the Lifshitz-van der Waals intermolecular interactions but it can interact by the acid-base forces with the adherent medium [27,28]. For the bipolar solids such as quartz, the surface tension results from both the Lifshitz-van der Waals and acid-base intermolecular interactions. The acid-base interactions originate mainly from the hydrogen bond formation called the hydrogen bond interactions.

The knowledge of the surface tension of solids and aqueous solution of the surfactants as well as of the relationship between the solid-solution interface tension ($\gamma_{SL}$), the solution and solid surface tension enables the explanation of the wetting properties of surfactants.

In 1805 Young studied the equilibrium state of the liquid drop settled on the solid surface and he stated that this equilibrium depends on the liquid and solid surface tension as well as the solid-liquid interface tension [1,20]. In turn, Dupre described this equilibrium state of the liquid drop settled on the solid surface by the thermodynamic equation, which is commonly called the Young equation [1,20]. From this equation it follows:

(1) $$\cos\theta =\frac{\gamma_{SV}-\gamma_{SL}}{\gamma_{LV}},\;$$

where $\gamma_{LV}$ is the surface tension of pure liquid or solution, $\gamma_{SV}$ is the solid surface tension and θ the contact angle.

The $\gamma_{SL}$ in Equation (1) can be replaced with the following relationship [27,28]:

(2) $$\gamma_{SL}=\gamma_{SV}+\gamma_{LV}-W_{a}=\gamma_{SV}+\gamma_{LV}-2\left(\sqrt{\gamma_{LV}^{LW}\gamma_{SV}^{LW}}+\sqrt{\gamma_{LV}^{+}\gamma_{SV}^{-}}+\sqrt{\gamma_{LV}^{-}\gamma_{SV}^{+}}\right),$$

where $W_{a}$ is the adhesion work of the liquid to the solid surface.

Introducing Equation (2) to Equation (1) we obtain:

(3) $$\cos\theta =\frac{-\gamma_{LV}+2\sqrt{\gamma_{LV}^{LW}\gamma_{SV}^{LW}}+2\sqrt{\gamma_{LV}^{+}\gamma_{SV}^{-}}+2\sqrt{\gamma_{LV}^{-}\gamma_{SV}^{+}}\;}{\gamma_{LV}}$$

In the case of apolar solids Equation (3) assumes the form:

(4) $$\cos\theta =\frac{-\gamma_{LV}+2\sqrt{\gamma_{LV}^{LW}\gamma_{SV}^{LW}}\;}{\gamma_{LV}}$$

Equations (3) and (4) are reliable if the layer of the liquid around the liquid drop does not change the solid surface tension [31].

The complete spreading of the liquid over the solid surface takes places if $2\sqrt{\gamma_{LV}^{LW}\gamma_{SV}^{LW}}+2\sqrt{\gamma_{LV}^{+}\gamma_{SV}^{-}}+2\sqrt{\gamma_{LV}^{-}\gamma_{SV}^{+}}=2\gamma_{LV}$ or for apolar solids $2\sqrt{\gamma_{LV}^{LW}\gamma_{SV}^{LW}}=2\gamma_{LV}$.

In order to prove whether the above mentioned conditions can be fulfilled for the complete spreading of the aqueous solution of biosurfactants and TX165 over the PTFE, PMMA and quartz surfaces, the adhesion work of biosurfactants, TX165 and water to these solid surfaces was calculated based on the components and parameters of the biosurfactants, TX165, water as well as the studied solids surface tension (Table 1 and Table 2). The calculations of the adhesion work were made for the surface active agents tail and head orientation toward the solid surface (Table 2). The calculation of $W_{a}$ of the biosurfactants and TX165 to PTFE indicates that in all cases its values are smaller than that of the cohesion work ($W_{C}$) and the spreading coefficient ($S_{L/S}$) [1] is smaller than zero (Table 2). This points out that it is not possible for the aqueous solutions of the biosurfactants and TX165 to spread over the PTFE surface completely. This conclusion is confirmed by the contact angle values presented in Figures S1–S3 in Supplementary Material. In the case of PMMA at the biosurfactants and TX165 molecules orientation by tail toward the PMMA surface, the $S_{L/S}$ values are positive, however for quartz they are positive at any orientation of the biosurfactants and TX165 molecules to its surface (Table 2). This fact indicates that in the case of the aqueous solutions of TX165, RL and SF at the appropriate concentration, complete spreading of these solutions over the PMMA and quartz surfaces should be observed. Unfortunately, the data presented in Figures S4 and S5 do not confirm this conclusion.

The adhesion work of TX165, RL and SF to the PTFE, PMMA and quartz surface as well as the cohesion work determine the limiting contact angle values of their aqueous solution. In such a case the biosurfactants and TX165 molecules are oriented perpendicularly towards the water-air interface and occupying the limiting area which is related to the geometrical size of the molecule. This limiting area at the water-air interface for TX165 is smaller than that for RL and SF but the SF limiting area is larger than the RL one (Table 3). At such packing of the biosurfactants and TX165 molecules the surface tension of their aqueous solution should be equal to the surfactants tail surface tension (Table 1). Unfortunately, the maximal packing $\left(\Gamma^{max}\right)$ of the TX165, RL and SF molecules corresponds to the minimal area $\left(A^{min}\right)$ occupied by one molecule which is larger than that of the limiting one $\left(A^{\infty }\right)$. Unexpectedly, the minimal surface area occupied by one molecule of TX165 and SF is larger than the contactable area of the molecule tail $\left(A_{T}^{cont}\right)$ at its parallel orientation towards the water-air interface. In the case of the RL these areas are comparable (Table 3). This fact suggests that the tail of biosurfactants and TX165 molecules in the saturated monolayer can be oriented parallel towards the water-air interface while the head perpendicularly and/or at an angle to this interface. In the case of TX165 there is the biggest difference between the minimal area occupied by its one molecule in the saturated monolayer at the water-air interface and that of the limiting area considering the used compounds. This can be reflected in the minimal value of the surface tension of the TX165 aqueous solution which is considerably greater than that for the RL and SF solutions (Figures S1–S3). The minimum value of the surface tension of the TX165 aqueous solution is significantly larger than that of the RL and SF solutions (Figures S1–S3) despite slight differences in the values of the surface tension of the TX165, RL and SF tails (Table 1). The differences between the minimal and limiting areas occupied by the biosurfactants and TX165 molecules in the adsorptive monolayer at the water-air interface result from that considering the attractive and repulsive interactions between their molecules. The hydrophilic part of the TX165 molecule is largely hydrated in the aqueous environment, and it can even acquire a positive charge [32,33]. For this reason, repulsive interactions between the TX165 molecules in the adsorptive monolayer at the water-air interface can occur, resulting in an increase in the surface area occupied by one TX165 molecule. In the case of biosurfactants, which can be treated as the 1:1 type electrolyte, due to the specific structure of their molecules head, the electrostatic repulsive interactions between the molecules in the monolayer can be neutralized by the hydrogen bonds formation between these molecules. Therefore, the biosurfactant molecules in the saturated monolayer are more closely packed than the TX165 which affects the surface tension of their solutions.

The behaviour of the biosurfactant and TX165 molecules at the PTFE-water interface is similar to that at the water-air one. This conclusion is supported by the Gibbs surface excess concentration of biosurfactants and TX165 values at the PTFE-water interface $\left(\Gamma_{SL}\right)$ calculated from the following equation [35]:

(5) $$\Gamma_{SL}=-\frac{C}{nRT}{\left[\frac{\partial \left(-\gamma_{LV}\cos\theta \right)}{\partial C}\right]}_{T}=-\frac{1}{2.303nRT}{\left[\frac{\partial \left(-\gamma_{LV}\cos\theta \right)}{\partial \log C}\right]}_{T},$$

where n is the parameter used in the Gibbs isotherm equation for determination of the surface excess concentration of a given surfactant, C is the surfactant concentration, R is the gas constant and T is the temperature.

The maximal values of $\Gamma_{SL}$ for TX165 $\left(2\times 10^{-6}\;\mathrm{mol}/m^{2}\right)$, RL $\left(1.98\times 10^{-6}\;\mathrm{mol}/m^{2}\right)$ and SF $\left(1.34\times 10^{-6}\;\mathrm{mol}/m^{2}\right)$ (Table 3) calculated from Equation (5) are similar to those of their Gibbs surface excess concentration at the water-air interface ($\Gamma_{LV})$ (TX165 $\left(2.12\times 10^{-6}\;\mathrm{mol}/m^{2}\right)$, RL $\left(2.01\times 10^{-6}\;\mathrm{mol}/m^{2}\right)$, and SF $\left(1.382\times 10^{-6}\;\mathrm{mol}/m^{2}\right)$ [5]) determined from the Gibbs isotherm equation. The similarity of $\Gamma_{SL}$ to $\Gamma_{LV}$ was also confirmed on the basis of the Lucassen-Reynders equation, which has the form [36]:

(6) $$\;\frac{\partial \left(\gamma_{LV}\cos\theta \right)}{\partial \gamma_{LV}}=\frac{\Gamma_{SV}-\Gamma_{SL}}{\Gamma_{LV}},\;$$

where $\Gamma_{SV}$ is the Gibbs surface excess concentration at the solid-air interface.

It proved that for PTFE the dependence of adhesion ($\gamma_{LV}\cos\theta$) and surface tension for the biosurfactants and TX165 can be described by the linear equation:

(7) $$\gamma_{LV}\cos\theta =a\gamma_{LV}+b,$$

where a and b are the constants. The constant a was close to −1 (Figure S4). In such case, according to Equation (6) $\Gamma_{SL}$ is close to $\Gamma_{LV}$ if the adsorption of the surfactants around the solution drop settled on the PTFE surface does not occur ($\Gamma_{SV}=0$). For the apolar solids such as PTFE whose surface tension results only from the Lifshitz-van der Waals intermolecular interactions if a=−1, the constant b fulfills the equation:

(8) $$b=W_{a}=\gamma_{LV}\left(\cos\theta +1\right)=2\sqrt{\gamma_{LV}^{LW}\gamma_{SV}^{LW}}.$$

As follows from Equation (8) if the contact angle is equal to zero, then on one hand, $\gamma_{LV}=\frac{b}{2}$ and on the other hand, $\gamma_{LV}=\sqrt{\gamma_{LV}^{LW}\gamma_{SV}^{LW}}$. From the linear relationship between $\gamma_{LV}\cos\theta$ and $\gamma_{LV}$ for PTFE, plotted based on the contact angle values for the aqueous solutions of TX165, RL and SF, it results that the constant b value is equal to 47.29 mN/m. This value is insignificantly higher than $W_{a}$ of water for PTFE (46.62 mJ/m^2^) (Table 2). This indicates that TX165 and biosurfactants reduce only the acid-base component of the water surface tension due to the adsorption of their molecules at the water-air interface. On the other hand, for the contact angle equal to zero, Equation (8) is not suitable since the $\gamma_{LV}^{LW}$ cannot be higher than $\gamma_{LV}$. Hence, it is not possible to determine the critical surface tension of PTFE wetting which corresponds to the contact angle equal to zero from Equation (8). The contact angle equal to zero can occur only if the surface tension of the liquid is equal to that of PTFE and this tension results only from the Lifshitz-van der Waals intermolecular interactions.

The evidence for the similar behaviour of the biosurfactants and TX165 molecules at the PTFE-water and water-air interface is also the fact that the isotherms of the surface tension of the aqueous solutions and the contact angle of these compounds can be described by the Szyszkowski equation with the similar constants related to the standard Gibbs free energy of adsorption ($\Delta G_{ads}^{0}$) (Figures S1–S3). The Szyszkowski equation for the isotherm of the solution surface tension can be expressed in the form [1]:

(9) $$\gamma_{0}-\gamma_{LV}=RTn\Gamma^{max}\ln\left(\frac{C}{a_{1}}+1\right),$$

where $\gamma_{0}$ is the water or the another solvent surface tension, $\Gamma^{max}$ is the maximal Gibbs surface excess concentration and $a_{1}$ is the constant which fulfills the equation [1]:

(10) $$a_{1}=\varpi \exp\frac{\Delta G_{ads}^{0}}{RT},$$

where ϖ is the number of water moles in 1 dm^3^.

If we assume that the thermodynamic equations can be applied for all interfaces, then the surface tension of solids does not depend on the surfactant concentration in the solution and the Szyszkowski equation can be written as:

(11) $$\gamma_{LV}\cos\theta -\gamma_{w}\cos\theta_{W}=RTn\Gamma^{max}\ln\left(\frac{C}{a_{1}}+1\right),$$

where $\gamma_{W}$ is the water surface tension and $\theta_{W}$ is the water contact angle on a given solid.

It appeared that it is possible to obtain almost the same values of $\Gamma^{max}$ and $a_{1}$ for TX165, RL and SF from Equations (10) and (11) (Figures S1–S3 and Table 3). This indicates that the $\Delta G_{ads}^{0}$ values calculated based on the $a_{1}$ constant from Equation (10) are similar (Table 3). Moreover, they are similar to those obtained from the Langmuir equation modified by de Boer, which has the following form (Table 3) [1,37]:

(12) $$\frac{A^{0}}{A-A^{0}}\exp\frac{A^{0}}{A-A^{0}}=\frac{C}{\varpi }\exp\left(\frac{-\Delta G_{ads}^{0}}{RT}\right),$$

where $A^{0}$ is the limiting area occupied by one molecule of the surfactant and A is the area occupied by one molecule of the surfactant in the monolayer at the interface corresponding to the given C value.

It should be mentioned that for the calculation of $\Gamma^{max}$ and $a_{1}$ from Equations (10) and (11) the values of C correspond to the monomeric form of the biosurfactants and TX165.

**Figure 1:**
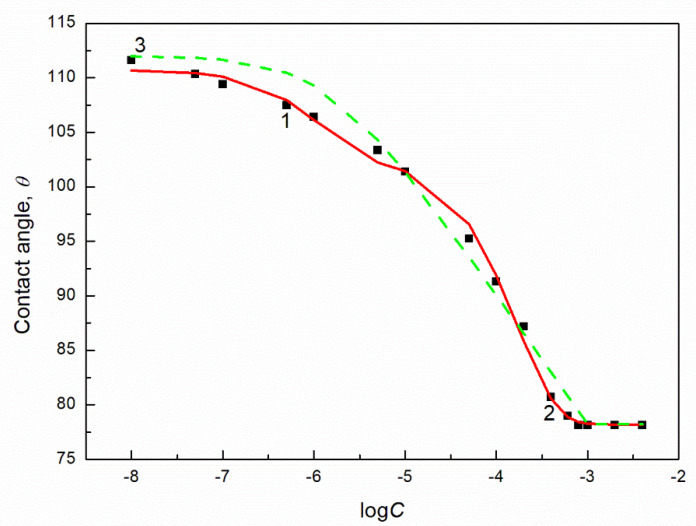
A plot of the TX165 aqueous solution contact angle (θ) on PTFE (curves 1–3) vs. the logarithm of TX165 concentration (C ). Curve 1 corresponds to the measured values, curve 2 to values calculated from the exponential function of the second order: $\theta =24.66595\exp\left(\frac{-C}{0.000170057}\right)+7.90722\exp\left(\frac{-C}{0.0000011843}\right)+78.1891$
curve 3 corresponds to the values calculated from Equations (9) and (11), respectively.

The isotherms of the contact angle of the aqueous solution of biosurfactants and TX165 on the PTFE surface can be described not only by the Szyszkowski equation but also by the exponential function of the second order (Figure 1 and Figure S1–S3). However, it is difficult to find the relationship between the constants in this function presented in figures and some physicochemical properties of solution components. These constants can be related to the components and parameters of the surface active agents and water surface tension similarly to the exponential function of the second order describing the isotherm of the surface tension of the aqueous solution of the surfactants [24].

The problem of PMMA and quartz wetting by the aqueous solutions of TX165, RL and SF is more complicated than in the case of PTFE. Contrary to PTFE, the surface tension of PMMA is higher than that of both the tail and head of TX165 and RL while in the case of quartz its surface tension is larger than that of the head and tail of all analyzed surface-active substances (Table 1). Moreover, the minimum surface tension of the aqueous solution of the biosurfactants and TX165 is smaller than that of PMMA and quartz (Figures S1–S3). According to van Oss et al. [27,28,29] it would seem that the aqueous solutions of biosurfactants and TX165 at the appropriate concentration should spread completely over the PMMA and quartz surfaces. Unfortunately, complete spreading over the PMMA and quartz surfaces did not occur for any of the solutions (Figures S5 and S6). It is possible that the reason for this fact is the migration of the biosurfactant and TX165 molecules on the PMMA and quartz surfaces. As the result of this migration, an adsorption layer is formed around the solution drop settled on the PMMA and quartz surfaces which changes their surface tension [38]. This can be confirmed by positive values of the spreading coefficient of the biosurfactants and surfactants on the PMMA and quartz surfaces (Table 2). The possibility of TX165, RL and SF adsorption around the settled solution drop on the PMMA and quartz surfaces is indicated by the curves showing the dependence between the adhesion and the solution surface tension (Figures S7 and S8). The slope of the curves for PMMA and quartz except for that obtained with the TX165 aqueous solution, is positive. In the case of PMMA the slope of the dependence between the adhesion and surface tension is negative for the RL and SF solution at their concentration higher than the critical micelle concentration (CMC) [39]. If the biosurfactants and TX165 are assumed not to adsorb on the PMMA and quartz surfaces around the drop of their solution, their negative adsorption at the solid-water interface takes place according to the Lucassen-Reynders equation [36]. This is impossible in practice. Probably due to the presence of the adsorption layer at the PMMA-air and quartz-air interfaces, the contact angle isotherm of the aqueous TX165, RL and SF solutions can not be described by Equation (11). It is interesting that using the difference between the contact angle for water and solution in the Szyszkowski equation instead of the layer pressure at the PMMA-water interface (PMMA(quartz)-water minus PMMA(solution)-solution interface tension), it was possible to describe the isotherm of the contact angle of the aqueous solution of TX165 for PMMA and quartz (Figures S7 and S8). Moreover, the values of the standard Gibbs free energy of adsorption calculated from Equation (10) based on the constant $a_{1}$ obtained in this way are close to the standard Gibbs free energy of adsorption values for TX165 at the PMMA-water and quartz-water interfaces calculated from the Langmuir equation modified by de Boer [1,37]. It should be mentioned that for the aqueous solution of RL it was impossible to describe the contact angle isotherm of its solution for PMMA and quartz as well as for the aqueous solution of SF for PMMA.

According to the Lucassen-Reynders equation, the positive slope of the curves obtained from the relationship between the adhesion and surface tension indicates that the adsorption of the surfactant at the solid-water interface is smaller than at the solid-air one [36]. This may be due to the very strong interactions of water molecules with the solid surface which is reflected in the $W_{a}$ values (Table 2), hindering the adsorption of surfactants. Due to these interactions of water molecules with the PMMA and quartz surfaces, there is smaller adsorption of TX165, RL and SF at the PMMA-water and quartz-water interfaces than at the water-air one (Table 3) [34]. The comparison of the minimal $\left(A^{min}\right)$ and limiting areas $\left(A^{\infty }\right)$ of TX165, RL and SF molecules occupied at these interfaces with the contactable area $\left(A^{cont}\right)$ of their molecules shows that they are oriented parallel to the PMMA and quartz surfaces (Table 3).

### 2.2. Contact Angle of Biosurfactants Mixtures with TX165

**Figure 2:**
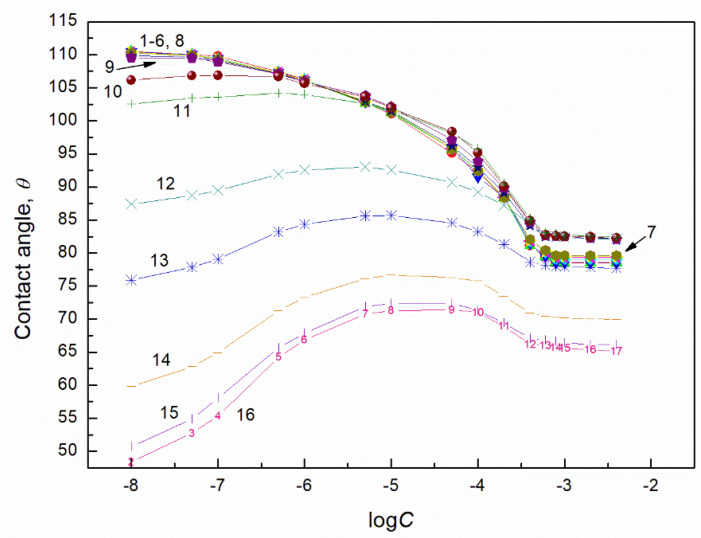
A plot of the contact angle (θ) of aqueous solution of TX165 + RL mixtures on the PTFE surface vs. the logarithm of the TX165 concentration (C). Curves 1–16 correspond to the constant RL concentration equal to 0.0002, 0.0005, 0.00125, 0.003, 0.00625, 0.01, 0.02, 0.05, 0.1, 0.5, 1, 5, 10, 20, 30 and 40 mg/dm^3^, respectively.

**Figure 3:**
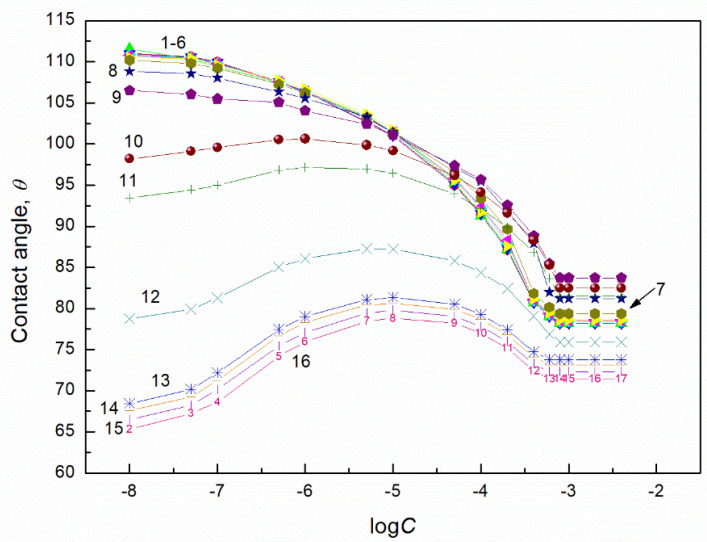
A plot of the contact angle (θ) of aqueous solution of TX165 + SF mixtures on the PTFE surface vs. the logarithm of the TX165 concentration (C). Curves 1–16 correspond to the constant SF concentration equal to 0.0002, 0.0005, 0.00125, 0.003, 0.00625, 0.01, 0.02, 0.05, 0.1, 0.5, 1, 5, 10, 20, 30 and 40 mg/dm^3^, respectively.

According to Equation (4) [27,28] in the case of apolar solids such as PTFE whose surface tension results only from the Lifshitz-van der Waals intermolecular interactions, the contact angle of surfactants aqueous solutions depends on the surface tension of the solution, the Lifshitz-van der Waals component of this tension and the PTFE surface tension. In turn, the surface tension of the aqueous solution depends on the components and parameters of the surface tension of all its constituents. Indeed, Equation (4) is applied for the calculation of the contact angle of liquids and solutions if the surface tension of apolar solids is not changed by the liquid vapour layer formed around the liquid drop settled on the solid surface. This proved that the liquid vapour having the surface tension larger than that of the solid does not change it. To explain the wetting properties of the TX165 + RL and TX165 + SF mixtures in the PTFE-aqueous solution of these mixtures-air system, the contact angle of hypothetical liquids on the PTFE surface, whose surface tension results only from the Lifshitz-van der Waals interactions, was calculated from Equation (4). Then it was assumed that the surface tension of hypothetical liquids results also from the Lifshitz-van der Waals intermolecular interactions and is equal to the Lifshitz-van der Waals components of water and tail of the biosurfactants and TX165. Taking into account the Lifshitz-van der Waals component for the water surface tension and that of RL, SF and TX165 head, the contact angle was calculated from Equation (4). As follows the contact angle for water, RL, SF and TX165 is equal to 42.57, 22.01, 35.86 and 23.32 degrees, respectively. These contact angle values indicate that it is impossible to achieve the complete spreading of the aqueous solution of the biosurfactants with the TX165 mixtures over the PTFE surface. This suggestion is confirmed by the contact angle values larger than zero (Figure 2 and Figure 3) as well as data presented in Figures S9 and S10. The minimal values of the surface tension of the aqueous solution of RL, SF and TX165 are higher than the Lifshitz-van der Waals component of water surface tension (Figures S1–S3) [40]. On the other hand, the Lifshitz-van der Waals component of the surface of the aqueous solution of the biosurfactants and TX165 does not depend on the solution concentration and is close to the Lifshitz-van der Waals component of water surface tension. This indicates that at the water-air interface the biosurfactants and TX165 do not reduce the LW component of water surface tension by their adsorption. However, they reduce its AB component but not to zero. On the other hand, the minimal surface tension of the aqueous solution of biosurfactants and TX165 mixtures is not smaller than the surface tension of the aqueous solution of individual mixture component. This may be the reason that the contact angle values of the studied mixtures aqueous solution are not smaller than those for the single biosurfactants and TX165 solutions (Figure 2, Figure 3 and Figure S1–S3).

It was found earlier that the surface tension of the biosurfactants with the TX165 mixtures depends on the contribution of particular components of the mixture to the reduction of water surface tension which is proportional to that of water surface tension by its single components [24]. As the contact angle isotherms of the aqueous solution of biosurfactants mixture for PTFE are similar to the surface tension isotherm, it can be possible to predict the contact angle of the mixture based on that of its particular components. In the other words, the contact angle of the aqueous solution of the surfactants mixture on the PTFE surface should depend on the sum of the products of the contact angle of the mixture components at the appropriate concentration and the fraction of the mixed layer at the PTFE-water interface (X). Thus, it can be written:

(13) $$\theta =\theta_{1}X_{1}+\theta_{2}X_{2},$$

where indices 1 and 2 refer to components 1 and 2 of the mixture.

This proved that the relationship between the adhesion and surface tension for all tested aqueous solutions of the biosurfactants with the TX165 mixture for PTFE is linear, and the line slope is close to −1. In that case, according to the Lucassen-Reynders equation (Equation (6)) [36], the concentration of the TX165 + biosurfactant mixture at the PTFE-water interface is similar to that at the water-air one. Assuming that the composition of the mixed layers at the PTFE-water and water-air interfaces is nearly the same, it is possible to calculate $X_{1}$ and $X_{2}$ from the following expression:

(14) $$X_{1}=\frac{\pi_{1}}{\pi_{1}+\pi_{2}}\mathrm{and}\;X_{2}=\frac{\pi_{2}}{\pi_{1}+\pi_{2}}$$

where $\pi_{1}$ and $\pi_{2}$ are the pressures of the monolayer at the water-air interface of the single components of the surfactant mixtures.

**Figure 4:**
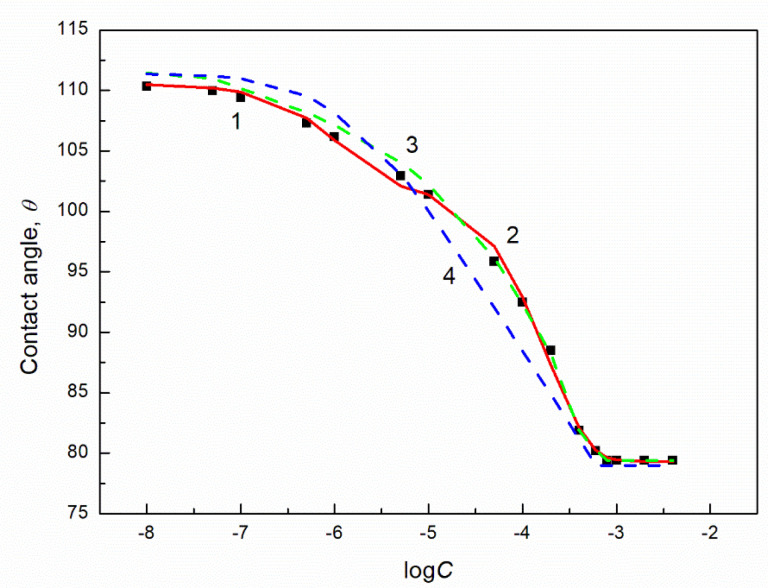
A plot of the contact angle (θ) of aqueous solution of TX165 + RL on PTFE at the constant RL concentration equal to 0.00625 vs. the logarithm of TX165 concentration (C). Points 1 correspond to the measured values, curves 2 correspond the values calculated form the exponential function of the second order:
$$\theta =23.28064\exp\left(\frac{-C}{0.00018624}\right)+7.93008\exp\left(\frac{-C}{0.00000117916}\right)+79.31736$$
and curves 3 and 4 correspond to values calculated from Equations (13) and (9), respectively.

Thus for the PTFE-solution drop-air systems in which the concentration of the biosurfactant and/or TX165 in the bulk phase of the solution corresponds to the unsaturated single monolayer at the water-air interface ($C_{unsat}$), the θ values calculated from Equation (13) based on Equation (14) are close to those measured (Figure 4 and Figure S11–S13). Unfortunately, at the concentration of one or two components of the TX165 + biosurfactant mixture higher than $C_{unsat}$ there is smaller agreement between the measured and calculated contact angle values than in the case mentioned above. Then the composition of the mixed layer at the PTFE-water interface can be slightly different from that at the water-air one, despite the equal total concentration of the surfactant mixture at these interfaces.

It should be emphasized that the contact angle isotherms for all studied solutions on the PTFE surface can be described by the exponential function of the second order (Figure 4 and Figure S11–S13 as an example). At the concentration of one or two components in the solution bulk phase higher than $C_{unsat}$ the isotherms of θ, on which the maxima are observed, can be described by the two exponential functions—one from the initial contact angle value to the maximal one and the other from the maximal value to the final one (Figures S11–S13). Similarly to the aqueous solution of RL, SF and TX165 the contact angle isotherm of the aqueous solution of the TX165 + biosurfactant mixtures on the PTFE surface can be described by the modified Szyszkowski equation (Equation (11)) (Figures S11–S13). Unfortunately, Equation (11) is fulfilled only for the solution in which the constant concentration of one component of the mixture is equal to $C_{unsat}$. It is worth noting that the calculated values of the standard Gibbs free energy of adsorption of individual components of the TX165 + biosurfactant mixture from equation (10) based on the obtained constant values from the Szyszkowski equation (Equation (11)) are similar to those calculated from the modified Langmuir equation for the water-air interface [1].

This suggests that the adsorption of the biosurfactants and TX165 mixtures at the PTFE-water interface affects only on the contribution of the acid-base interaction to the PTFE-water interface tension. This suggestion is confirmed by the relationship between the adhesion and the surface tension (Figure S14). The dependence shows that not only the adsorption of the TX165 + biosurfactant mixture at the PTFE-water and water-air interfaces is similar but also that the adhesion work of the solution to the PTFE surface does not depend on the mixture composition and concentration. The values of the adhesion work of solution mixtures are close to that of water to the PTFE surface.

Some authors [41,42] suggest that in the case of the linear relationship between the adhesion and surface tension with the slope equal to −1, the value equal to half of the work adhesion obtained from this relationship is equal to the critical surface tension of solid wetting. In the case of the aqueous solution of the biosurfactant and TX165 mixtures the value of the half of the adhesion work of the solution to the PTFE surface is equal to 23.75 mJ/m^2^. According to the above considerations it is impossible to acquire the complete spreading of the studied mixture over the PTFE surface which is further confirmed by the isotherms of the solution contact angle.

**Figure 5:**
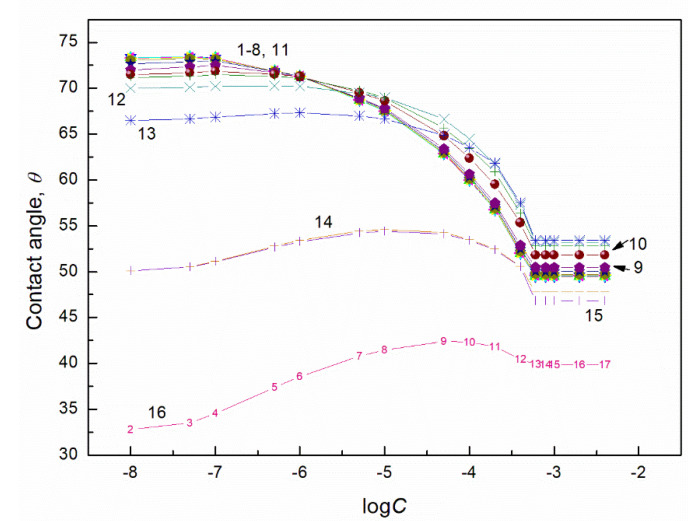
A plot of the contact angle (θ) of aqueous solution of TX165 + RL mixtures on the PMMA surface vs. the logarithm of the TX165 concentration (C). Curves 1–16 correspond to the constant RL concentration equal to 0.0002, 0.0005, 0.00125, 0.003, 0.00625, 0.01, 0.02, 0.05, 0.1, 0.5, 1, 5, 10, 20, 30 and 40 mg/dm^3^, respectively.

**Figure 6:**
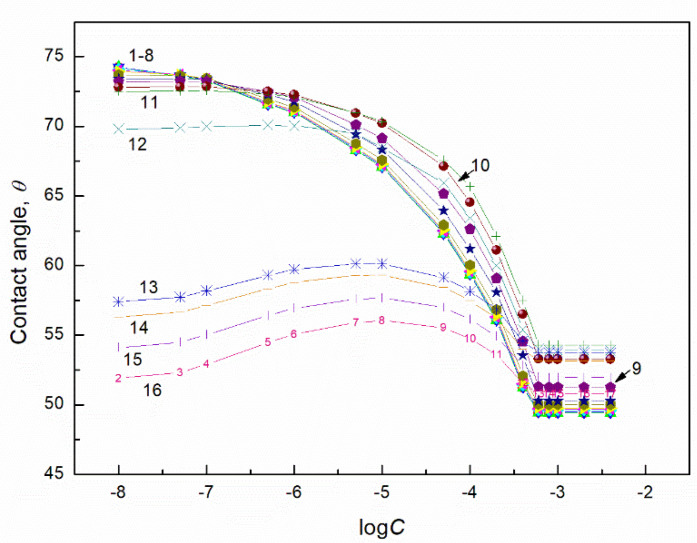
A plot of the contact angle (θ) of aqueous solution of TX165 + SF mixtures on the PMMA surface vs. the logarithm of the TX165 concentration (C). Curves 1–16 correspond to the constant SF concentration equal to 0.0002, 0.0005, 0.00125, 0.003, 0.00625, 0.01, 0.02, 0.05, 0.1, 0.5, 1, 5, 10, 20, 30 and 40 mg/dm^3^, respectively.

**Figure 7:**
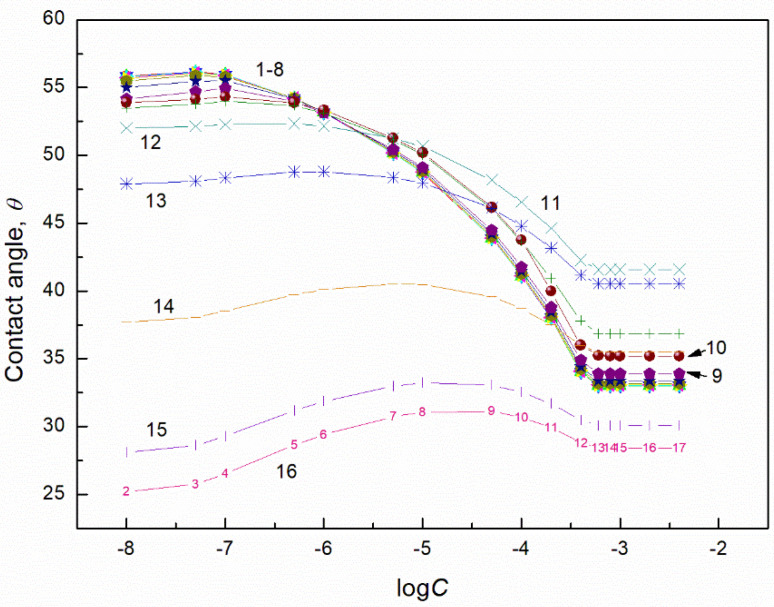
A plot of the contact angle (*θ*) of aqueous solution of TX165 + RL mixtures on the quartz surface vs. the logarithm of the TX165 concentration (*C*). Curves 1–16 correspond to the constant RL concentration equal to 0.0002, 0.0005, 0.00125, 0.003, 0.00625, 0.01, 0.02, 0.05, 0.1, 0.5, 1, 5, 10, 20, 30 and 40 mg/dm^3^, respectively.

**Figure 8:**
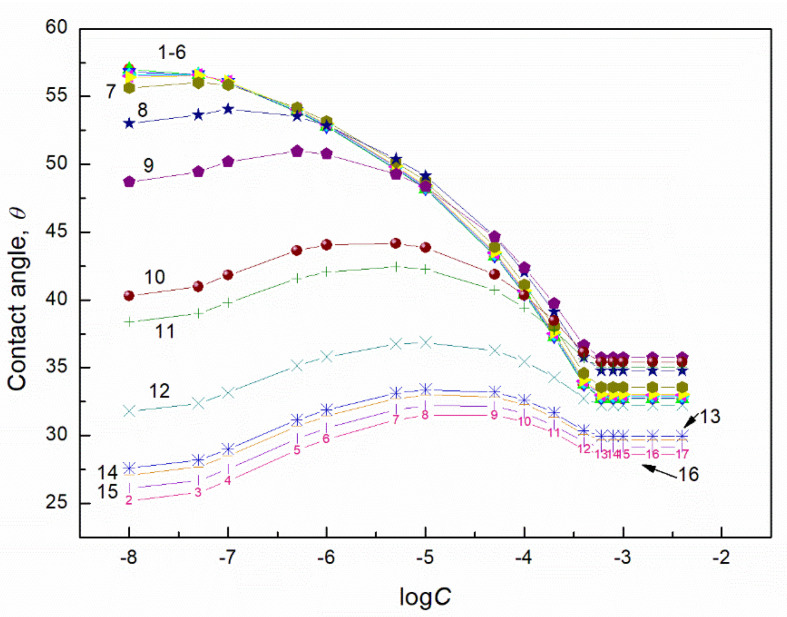
A plot of the contact angle (θ) of aqueous solution of TX165 + SF mixtures on the quartz surface vs. the logarithm of the TX165 concentration (C). Curves 1–16 correspond to the constant SF concentration equal to 0.0002, 0.0005, 0.00125, 0.003, 0.00625, 0.01, 0.02, 0.05, 0.1, 0.5, 1, 5, 10, 20, 30 and 40 mg/dm^3^, respectively.

The wetting process of PMMA and quartz by the aqueous solution of TX165 + RL and TX165 + SF is more complicated than that of PTFE. As mentioned above the surface tension of the surfactants tail is smaller than that of PMMA and quartz. In the case of the surfactant head surface tension, its value is insignificantly higher than the surface tension of PMMA only for SF. Thus, theoretically it is possible to obtain the complete spreading of the aqueous solution of the TX165 + RL and TX165 + SF mixtures over the PMMA and quartz surface. However, no complete spreading over the surface for the mentioned solids was obtained (Figure 5, Figure 6, Figure 7 and Figure 8 and Figure S15–S18).

**Figure 9:**
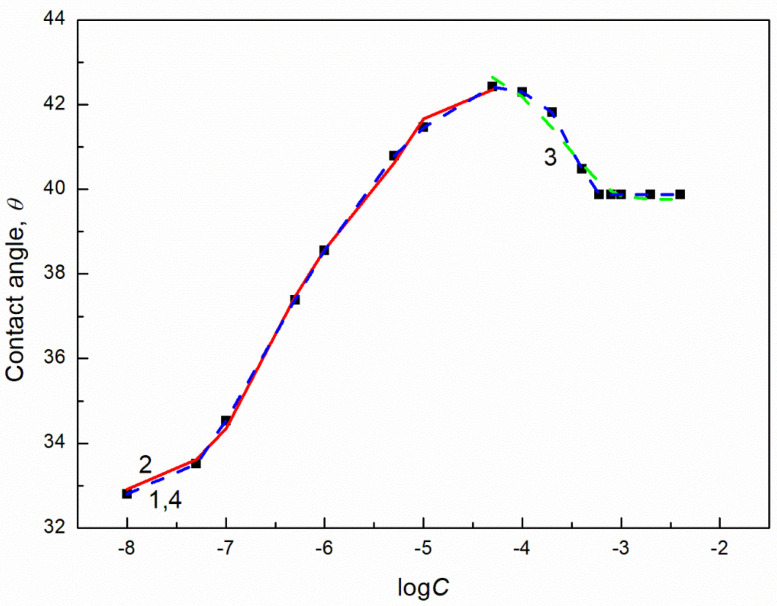
A plot of the contact angle (θ) for TX165 + RL for PMMA at the constant RL concentration equal to 40 mg/dm^3^ vs. the logarithm of TX165 concentration (C). Points 1 correspond to the measured values, curve 2 corresponds the values calculated from two exponential functions of the second order:$\left(\theta =-5.28316\exp\left(\frac{-C}{0.0000028661}\right)-4.34766\exp\left(\frac{-C}{0.0000054396}\right)+42.35076\right)$,$\left(\theta =1.73099\exp\left(\frac{-C}{0.00028}\right)+1.73099\exp\left(\frac{-C}{0.00028}\right)+39.75775\right)$, and curves 3 and 4 correspond to values calculated from Equations (13) and (9), respectively.

As mentioned above the values of the spreading coefficient of the biosurfactants and TX165 over the PMMA and quartz surface are positive independent of the way of molecules orientation towards their surface (except for the TX165 head-PMMA system) (Table 2). Thus, it is possible that the biosurfactants and TX165 can penetrate from the solution drop settled on the quartz or PMMA surface, changing the surface tension of PMMA and quartz around the drop. In that case the surface tension of the PMMA and quartz is variable as a function of the aqueous solution of the biosurfactants and TX165 mixtures concentration and composition. This suggestion is confirmed by the relationships between the adhesion and surface tension for both PMMA and quartz (Figures S19–S22). The slope of the curves of these relationships changes as a function of the composition and concentration of the solution of the studied mixtures. It depends also on the type of the solids and for PMMA it changes even from negative to positive values. This indicates that the slope of the curve representing the dependence between the adhesion and surface tension for the solution of TX165 + biosurfactant mixture at the same composition and concentration is different for PMMA and quartz (Figures S19–S22). According to the Lucassen-Reynders equation (Equation (6)) the changes of the slope of this dependence as a function of the composition and the concentration of the solution of the biosurfactant and TX165 mixture are due to those in the correlation between the adsorption of this mixture at the solid-air and solid-liquid interfaces. This relation influences on the isotherm of the contact angle. Similarly to PTFE, the maxima on the isotherms of the contact angle of the aqueous solution of the TX165 + RL and TX165 + SF mixtures on the PMMA and quartz surfaces are present for the solutions whose concentration of one and/or two mixture components is higher than $C_{unsat}$ (Figures S15–S18). These maxima can be explained based on the contact angle isotherms calculated from Equation (13) (Figure 9 and Figure S23–S26). 

In most cases the theoretical isotherms of the contact angle are similar to those obtained from the measured contact angle even at the solution concentration of particular components of the mixture higher than $C_{unsat}$ (Figure 9 and Figure S23–S26). The agreement between the isotherm obtained from the calculations and measurements of the contact angle suggests that the particular components contribution to the contact angle is proportional to the reduction of water surface tension. Moreover the composition of the surface mixed monolayer at the solid-air and the solid-water interfaces is similar despite different concentrations. If there is no such agreement, there can be differences in the composition of the interface layers at the solid-air and solid-water interfaces. It should be mentioned that all isotherms of the contact angle of the aqueous solution of TX165 + RL and TX165 + SF on the PMMA and quartz surface can be described by the exponential function of the second order (Figure 9 and Figure S23–S26). In the case of the solution at the concentration of one or two components of a given mixture higher than $\;C_{unsat}$, the isotherms of the contact angle were described by the two exponential functions: one in the concentration range of the component at the variable concentration with its value from zero to that corresponding to the maximal value and the other one from the maximum to the limited concentration value. In the case of the solutions at the constant concentration smaller than $\;C_{unsat}$, the contact angle isotherm can be also described by the modified Szyszkowski equation (Figure 9 and Figure S23–S26). The values of the standard Gibbs free energy of adsorption obtained from this equation are higher than those obtained for the individual components of the mixture.

### 2.3. Composition and Concentration of the Mixed Layer at the Solid-Air and Solid-Water Interfaces

The dependence between the adhesion and surface tension of the aqueous solutions of TX165 + RL and TX165 + SF both at the constant biosurfactants concentration and variable TX165 and vice versa for PTFE can be expressed by one linear dependence (Figure S14). Its slope is close to −1. Thus according to the Lucassen-Renders equation [35] (Equation (6)) the Gibbs surface excess concentration of the biosurfactants and TX165 mixture at the PTFE-water interface is close to that at the water-air one. As a matter of fact, there should be satisfied the condition that the surface tension of PTFE does not depend on the concentration and composition of the biosurfactants and TX165 mixtures. The constant *b* in the linear equation describing the dependence between the adhesion and surface tension is close to the adhesion work of water to the PTFE surface (Table 2). This means that 2$\sqrt{\gamma_{W}^{LW}\gamma_{W}^{LSV}}$ is close to $\gamma_{LV}\left(\cos\theta +1\right)$. Thus it follows that the mixtures of the biosurfactants with TX165 do not reduce the Lifshitz-van der Waals component of the water surface tension (Table 1) and that the surface tension of PTFE does not depend on the concentration and composition of the TX165 + RL and TX165 + SF mixtures. The linear dependence between the adhesion and surface tension at the slope equal to −1 does not prove that despite the same concentration of the biosurfactants mixture with TX165 at the water-air and the PTFFE-water interfaces, the composition of the mixed monolayer is the same. For this reason the Gibbs surface excess concentration of the biosurfactants and TX165 mixtures at the PTFE-water interface was calculated from Equation (5). However, it was possible to calculate the Gibbs surface excess concentration at the PTFE-water interface only in the range of the constant concentration value of one component of the mixture smaller than $C_{unsat}$. For such a case the dependence between the adhesion tension and the variable concentration of the other component of the mixture can be expressed by the exponential function of the second order. The maximal excess concentration of the biosurfactant or TX165 was determined from the linear dependence between the concentration logarithm and the adhesion tension. In the case of the constant concentration of one component of the mixture and variable of the other one at which the maxima on the contact angle isotherm are present, the Gibbs surface excess concentration of the biosurfactants and TX165 was calculated from the following expression:

(15) $$\Gamma_{1}^{max}X_{1}+\Gamma_{2}^{max}X_{2}=\Gamma_{12}.$$

It seems reasonable to assume that the contribution of the particular component mixture to its total concentration at the PTFE-water interface similarly to the contact angle should be proportional to that of this component in the water surface tension reduction. Thus for the calculation of $\Gamma_{12}$ the values of $X_{1}$ and $X_{2}$ determined in the above mentioned way were used in Equation (15) (Figure S27).

As follows from the comparison of the Gibbs surface excess concentration of the biosurfactants and TX165 at the PTFE-water and the water-air interfaces the composition of its mixed monolayer at the PTFE-water interface is somehow different from that at the water-air one [24] (Figures S28–S31). It should be remembered that the adsorption mechanism for the same surfactant at the PTFE-water and water-air interfaces is different. In the adsorption at the PTFE-water interface the hydrophobic interactions between PTFE and the surfactant tail play a very important role. They depend on the PTFE-water and the tail-water interface tension and the contactable area of the surfactant tail. Hence, these differences in the composition of the mixed monolayer at the PTFE-water and water-air interfaces are likely to take place. Nevertheless, as indicated by the values of the Gibbs standard free energy of adsorption of the biosurfactants and TX165, calculated from both the Langmuir equation modified by de Boer and based on the modified Szyszkowski equation, the tendency for the biosurfactant and TX165 to adsorb at the PTFE-water interface is similar to that at the water-air one (Table 4) [24].

In the case of the monopolar PMMA and bipolar quartz the determination of the biosurfactants and TX165 adsorption is more complicated than at the PTFE-water interface.

Based on the relationship between the adhesion and surface tension of the aqueous solution of the biosurfactants and TX165 mixture for PMMA and quartz, the amount of the adsorbed mixture at the solid-water interface cannot be deduced contrary to PTFE. There is no linear dependence between the adhesion and surface tension for PMMA and quartz. Moreover, the curves representing this dependence can assume the positive slope which would suggest the negative adsorption (Figures S19–S22). The values of adhesion work obtained from the van Oss at al. [27,28,29] and Young-Dupre equations [1,20] for all studied solutions to the PMMA and quartz surface indicate that $2\sqrt{\gamma_{LV}^{LW}\gamma_{SV}^{LW}}+2\sqrt{\gamma_{LV}^{+}\gamma_{SV}^{-}}+2\sqrt{\gamma_{LV}^{-}\gamma {\;}_{SV}^{+}}>\gamma_{LV}\left(\cos\theta +1\right)$. This means that the surface tension of PMMA and quartz changes as a function of composition and concentration of the biosurfactants and TX165 mixture. This satisfies the equation:

(16) $$2\sqrt{\gamma_{LV}^{LW}\gamma_{SV}^{LW}}+2\sqrt{\gamma_{LV}^{+}\gamma_{SV}^{-}}+2\sqrt{\gamma_{LV}^{-}\gamma {\;}_{SV}^{+}}-\gamma_{LV}\left(\cos\theta +1\right)=\pi .$$

As follows at the first approximation $\pi \approx \frac{\gamma_{W}-\gamma_{LV}}{2}$. Taking into account Equation (16) and the Young equation it was possible to determine the PMMA-solution and quartz-solution interface tension as well as the PMMA-surface layer and quartz-surface layer interface tensions. Then the Gibbs surface excess concentrations of SF, RL and TX165 at these interfaces could be determined using the following equations:

(17) $$\Gamma_{SV}=-\frac{C}{nRT}{\left[\frac{\partial \gamma_{SV}}{\partial C}\right]}_{T}=-\frac{1}{2.303nRT}{\left[\frac{\partial \gamma_{SV}}{\partial \log C}\right]}_{T},$$

(18) $$\Gamma_{SL}=-\frac{C}{nRT}{\left[\frac{\partial \gamma_{SL}}{\partial C}\right]}_{T}=-\frac{1}{2.303nRT}{\left[\frac{\partial \gamma_{SL}}{\partial \log C}\right]}_{T}.$$

Based on Equations (17) and (18) it was possible to calculate the $\Gamma_{SV}$ and $\Gamma_{SL}$ values at the constant concentration of one mixture component smaller than its $C_{unsat}$. In the other case the $\Gamma_{SV}$ and $\Gamma_{SL}$ values were determined from expression (15) (Figures S32–S35).

It appeared that the values of $\Gamma_{SV}$ and $\Gamma_{SL}$ are significantly smaller than that of $\Gamma_{LV}$ [24] (Figures S32–S35). As follows from the single surfactants layer at the PMMA-air, PMMA-water, quartz-air and quartz-water interfaces the molecules of biosurfactants and TX165 in the mixed layer are oriented parallel towards the interface. However, the Gibbs surface excess concentration of TX165 in the saturated mixed monolayer at the PMMA-air, quartz-air as well as the PMMA-water and quartz-water interfaces is higher than that for the biosurfactants. This may result from three cases: (1) the oxyethylene groups in the TX165 molecule can be connected with hydrogen ions, (2) active electric interactions occur between the oxyethylene group and hydrogen ions, (3) interactions of particularly negatively charged quartz surface and/or –CO groups on the PMMA surface take place. On the other hand, one molecule of biosurfactants can remove more water molecules from the interface than one molecule of TX165 (Table 3). The relationship between the values of $\Gamma_{SV}$ and $\Gamma_{SL}$ explains the slope of the curves representing the dependence between the adhesion and surface tension (Figures S19–S22, S36 and S37). In the case of quartz this slope is positive for all studied solutions because $\Gamma_{SV}$ is higher than $\Gamma_{SL}$. This may result from the fact that the heads of the biosurfactants and TX165 molecules interact strongly with the water ones (Table 2). The interactions of the biosurfactants and TX165 molecules with the PMMA and quartz surface through the water phase depend on the solid-tail, solid-head as well as the water-tail and water-head interactions and the contactable area of tail and head [43]. These interactions are associated with the interface tension. As the water-head of the biosurfactant and/or the water-head of TX165 interface tension is negative, it reduces the possibility of surfactants to adsorb at the PMMA-water and quartz-water interfaces compared to the PMMA-air and quartz-air interfaces. This is more evident in the case of the systems including quartz instead of PMMA. Despite smaller adsorption of the biosurfactants and TX165 mixture at the PMMA-air, quartz-air, PMMA-water and quartz-water interfaces than at the water-air one, the values of standard Gibbs free energy of adsorption calculated from the Langmuir equation modified by de Boer Equation (12) are similar to those of the standard Gibbs free energy of adsorption at the water-air interface. It is worth mentioning that the values of the standard Gibbs free energy of adsorption determined based on the Szyszkowski equation are close to those calculated from the Langmuir equation (Table 4). It should be pointed out that the values of $\Gamma_{SV}$ and $\Gamma_{SL}$ calculated from the modified Szyszkowski equation in which instead of the difference between the interface tension of water and solution that between the contact angle for water and solution were applied on the PMMA and quartz surface. The difference between the contact angle of water on the surfaces of PMMA and quartz and the solution is close to that between the PMMA (quartz)-water and PMMA (quartz)-solution and/or between the surface tension of PMMA (quartz)-air in the presence of the water drop and the solution (Figure S38). 

## 3. Materials and Methods

Triton X-165 (p-(1,1,3,3-tetramethylbutyl)-phenoxypolyoxyethylene glycol) of purity over 99% was purchased from FLUKA. R-95 Rhamnolipid (95%) (RL) and surfactin (≥98%) (SF) were purchased from Sigma-Aldrich. Nonionic TX165 and biosurfactants were used without further purification. Four series of the aqueous solutions of TX165 with the biosurfactant mixture were prepared for the contact angle measurements on the PTFE, PMMA and Q surface. The first and second series included the aqueous solutions of the mixture of TX165 with RL and SF, in which the biosurfactant concentration was constant and that of TX165 was variable. The third and fourth series included the solutions of the mixture of TX165 with RL and SF in which the concentration of TX165 was constant and concentration of the biosurfactant changed. The range of biosurfactants concentration in all series of solutions was from 0 to 40 mg/dm^3^, and that of TX165 from 0 to 4 × 10^−3^ mole/dm^3^. The range of the biosurfactants and TX165 concentration in the aqueous solution included the value of CMC of the given surface active agent. The water used for preparation of all solutions was doubly distilled and deionized (Destamat Bi18E). The internal specific resistance of water was 18.2 × 10^6^ Ω⋅m. Before the solution preparation the water purity was additionally controlled by the surface tension measurements. 

The polymers used for the contact angle measurements were obtained from Mega-Tech, Poland and the quartz solids from Conductance, Poland. Before the contact angle measurements the surfaces of the polymers and quartz plates were prepared according to the procedure described earlier [34], which was used twice for each plate. Then the plates were dried and placed in a desiccator with the molecular sieve.

The advancing contact angle (θ) for the aqueous solutions of TX165 + biosurfactant mixture on the PTFE, PMMA and quartz surfaces was measured by the sessile drop method at 293 ± 0.1 K using the DSA30 measuring system (Krüss, Hamburg, Germany) with a thermostated chamber. For determination of the contact angle values both tangential and circle fittings were used. The procedure for measuring the contact angle was described in detail previously [35]. The drop volume of 7 µL was used for all contact angles measurements. The contact angle measurements for each solution on the PTFE, PMMA and quartz surfaces were repeated 10 times. The standard deviation of the contact angle values was related to the concentration of the biosurfactants and TX165 in the solution. At the concentration corresponding to the unsaturated mixed monolayers at the water-air interface the standard deviation was smaller than 1.5° but at that corresponding to the saturated mixed monolayer was larger, being close to 2°.

## 4. Conclusions

Based on of the measurements of the contact angle and the thermodynamic analysis of the obtained results, a number of conclusions can be drawn.

In no case the aqueous solutions of the TX165 and biosurfactants mixtures spread completely over the PTFE, PMMA and quartz surface. In the case of PMMA and quartz this results from the fact that around the solution drop settled on the PMMA and quartz surface the mixed biosurfactant and TX165 layer is formed, changing their.

The contact angle isotherms of the aqueous solution of TX165 with RL and SF can be described by the exponential function of the second order even when the maxima are present on them. In that case there can be used two exponential functions: one for the solution concentration from zero to that corresponding to the maximum and the other one from the concentration corresponding to the maximum and the final concentration.

In most cases the contact angle isotherms can be predicted from those of the surface tension of the aqueous solution of individual TX165, RL and SF.

In the PTFE-aqueous solution of the biosurfactant mixture with the TX165 drop-air system, the contact angle changes as a result of the acid-base component of water surface tension reduction due to its adsorption at the water-air interface.

In some cases it is possible to describe the isotherms of the contact angle by the Szyszkowski equation using: (1) the difference between the adhesion tension of water and solution for the PTFE-solution system, (2) the difference between the contact angle for water and solution in the PMMA-air, PMMA-solution, quartz-air and quartz-solution systems.

The adsorption of the biosurfactant + TX165 mixture at the PTFE-water interface is comparable with that at the water-air one, but there are some differences in the surface layers composition.

The adsorption of the biosurfactant mixtures with TX165 at the PMMA-air, PMMA-water, quartz-air and quartz-water interfaces is smaller than at the water-air one. In the case of the systems including quartz, the adsorption of the biosurfactant + TX165 mixture at the quartz-air interface is larger than that at the quartz-water interface.

The standard Gibbs free energy of adsorption of biosurfactant + TX165 mixture at the PTFE-water, PMMA-air, PMMA-water, quartz-air and quartz-water interfaces is comparable to that at the water-air interface.

## Figures and Tables

**Table 1:** The values of the Lifshitz-van der Waals ($\gamma^{LW}$) and acid-base ($\gamma^{AB}$) components as well as electron-acceptor ($\gamma^{+}$) and electron-donor ($\gamma^{-}$) parameters of water, TX165, rhamnolipid and surfactin head and tail as well as PTFE, PMMA and quartz surface tension (*γ*).

Substance	Components and Parameters [mN/m]				γ [mN/m]	Refs.
	$\gamma^{LW}$	$\gamma^{AB}$	$\gamma^{+}$	$\gamma^{-}$		
**SF (head)**	34.25	8.55	0.37	49.39	42.80	[24,34]
**SF (tail)**	24.70	0.00	0.00	0.00	24.70	[24,34]
**Water**	26.85	45.95	22.975	22.975	72.80	[24,34]
**RL (head)**	35.38	3.01	0.04	56.74	38.39	[24,34]
**RL (tail)**	21.80	0.00	0.00	0.00	21.80	[24,34]
**TX165 (head)**	27.70	8.14	0.33	50.20	35.84	[24,34]
**TX165 (tail)**	22.00	0.00	0.00	0.00	22.00	[24,34]
**PTFE**	20.24	0.00	0.00	0.00	20.24	[35]
**PMMA**	41.28	0.00	0.00	7.28	41.28	[35]
**Quartz**	38.07	9.63	1.61	14.36	47.70	[35]

**Table 2:** The values of adhesion work ($W_{a}$), solid-liquid interface tension ($\gamma_{SL}$) calculated from Equations (1) and (2), respectively and the values of spreading coefficient ($S_{L/S}$).

Substrates	PTFE			PMMA			Quartz		
	$W_{a}$	$\gamma_{SL}$	$S_{L/S}$	$W_{a}$	$\gamma_{SL}$	$S_{L/S}$	$W_{a}$	$\gamma_{SL}$	$S_{L/S}$
**SF (head)**	52.66	10.38	−32.94	78.48	5.60	−7.12	94.66	−4.16	9.06
**SF (tail)**	44.72	0.22	−4.68	63.86	2.12	14.46	61.33	11.07	11.93
**Water**	46.62	46.42	−98.98	92.45	21.63	−53.15	112.43	8.07	−33.17
**RL (head)**	53.52	5.11	−23.26	77.51	2.16	0.73	94.03	−7.94	17.25
**RL (tail)**	42.01	0.03	−1.59	60	3.08	16.40	57.62	11.88	14.02
**TX165 (head)**	47.36	8.72	−24.32	70.73	6.39	−0.95	87.28	−3.74	15.60
**TX165 (tail)**	42.20	0.04	−1.80	60.27	3.01	16.27	57.88	11.82	13.88

**Table 3:** The values of the standard Gibbs free energy of the adsorption Δ($G_{ads}^{0}$), Gibbs excess concentration (maximal—$\Gamma^{max}$ and limiting—$\Gamma^{\infty }$) and the area occupied by TX165, RL and SF at the water-air (W-A), PTFE-water (PTFE-W), PMMA-water (PMMA-W) and quartz-water (Q-W) interfaces (minimal—$A^{min}$ and limiting—$A^{\infty }$) as well as the contactable area of the tail $\left(A_{T}^{cont}\right)$ and head $\left(A_{H}^{cont}\right)$ of these surfactants molecule.

	Δ $G_{ads}^{0}[\mathrm{kJ}/\mathrm{mol}]$					
	TX 165		RL		SF	
**Interface**	**Equation (12) ^1^**	**Equation (10)**	**Equation (12) ^1^**	**Equation (10)**	**Equation (12) ^1^**	**Equation (10)**
**W-A**	−44.00	−44.23	−42.57	−44.79	−47.37	−51.53
**PTFE-W**	−43.89	−42.53	−42.70	−44.59	−51.54	−52.15
**PMMA-W**	−44.58	−44.11	−43.31	-	−49.52	-
**Q-W**	−43.58	−42.53	−43.02	-	−50.23	-
**Gibbs surface excess concentration**, Γ **(×10^−6^ mol/m^2^)**						
**Interface**	$\Gamma^{max}$	$\Gamma^{\infty }$	$\Gamma^{max}$	$\Gamma^{\infty }$	$\Gamma^{max}$	$\Gamma^{\infty }$
**W-A**	2.12	4.65	2.01	2.403	1.382	1.782
**PTFE-W**	2.10	4.65	1.98	2.28	1.34	1.75
**PMMA-W**	1.27	2.80	0.71	0.91	0.55	1.10
**PMMA-A**	1.00	2.80	0.98	1.20	0.64	1.10
**Q-W**	0.50	1.03	0.34	0.47	0.51	0.87
**Q-A**	0.91	1.20	0.69	0.91	0.67	0.87
**Occupied area [Å^2^]**						
	$A^{min}$	$A^{\infty }$	$A^{min}$	$A^{\infty }$	$A^{min}$	$A^{\infty }$
**W-A**	78.32	35.70	82.60	69.09	120.38	93.14−120.24
**PTFE-W**	79.06	35.70	82.80	72.82	123.90	93.14−120.24
**PMMA-W**	130.73	59.30	233.85	182.45	301.87	150.94
**PMMA-A**	166.03	59.30	169.41	138.36	259.42	150.94
**Q-W**	332.06	161.19	488.32	353.26	325.55	190.84
**Q-A**	182.45	138.36	240.62	132.45	247.81	190.84
**Contactable area,** $A^{cont}$ **[Å^2^]**						
	**TX165**		**RL**		**SF**	
**Surfactant**	**Tail**	**Head**	**Tail**	**Head**	**Tail**	**Head**
	61.12	100.61	87.25	72.13	75.95	255.56

^1^ The all data concerning the water-air interface (W-A) calculated from Equation (12) were taken from the literature [38].

**Table 4:** The values of standard Gibbs free energy of adsorption of TX165, RL and SF ($\Delta G_{ads}^{0}$) at the PTFE-water, PMMA –water, quartz-water, PMMA-air and quartz-air interfaces calculated from Equations (10) and (12) respectively.

$\Delta G_{ads}^{0}\;[\mathrm{kJ}/\mathrm{mol}]$										
	PTFE-W		PMMA-A		PMMA-W		Q-A		Q-W	
Mixture	Equation (12)	Equation (10)	Equation (12)	Equation (10)	Equation (12)	Equation (10)	Equation (12)	Equation (10)	Equation (12)	Equation (10)
**TX165 + RL** **(const RL)**	−43.63	−42.91	−42.89	−42.11	−42.90	−41.91	−35.79	-	−44.16	−43.44
**TX165 + SF** **(const SF)**	−44.17	−43.84	−43.47	−42.74	−44.05	−43.25	−46.35	−45.15	−46.18	−46.00
**TX165 + RL** **(const TX165)**	−42.13	−41.51	−42.66	−42.15	−45.36	−45.14	−46.07	−45.81	−48.51	−48.01
**TX165 + SF** **(const TX165)**	−49.42	−48.15	−48.71	−49.05	−49.33	−49.11	−51.16	−50.25	−52.64	−51.13

## Data Availability

Not applicable.

## Supplementary Materials

The following supporting information can be downloaded at: https://www.mdpi.com/article/10.3390/molecules27154706/s1, Figure S1: A plot of the TX165 aqueous solution contact angle (θ) surface tension ($\gamma_{LV}$) (curves 1–3) and contact angle (θ) on PTFE (curves 1′–3′) vs. the logarithm of TX165 concentration (C). Curves 1 and 1′ correspond to the measured values, curves 2 and 2′ to values calculated from the exponential function of the second order, curves 3 and 3′ correspond to the values calculated from Equations (9) and (11), respectively; Figure S2: A plot of the RL aqueous solution surface tension ($\gamma_{LV}$) (curves 1–3) and contact angle (θ) on PTFE (curves 1′–3′) vs. the logarithm of RL concentration (C). Curves 1 and 1′ correspond to the measured values, curves 2 and 2′ to values calculated from the exponential function of the second order, curves 3 and 3′ correspond to the values calculated from Equations (9) and (11), respectively; Figure S3: A plot of the SF aqueous solution surface tension ($\gamma_{LV}$) (curves 1–3) and contact angle (θ) on PTFE (curves 1′–3′) vs. the logarithm of SF concentration (C). Curves 1 and 1′ correspond the measured values, curves 2 and 2′ to values calculated from the exponential function of the second order, curves 3 and 3′ correspond to the values calculated from Equations (9) and (11), respectively; Figure S4: A plot of the contact angle (θ) of aqueous solution of TX165 (curves 1, 1′ and 1″), RL (curve 2) and SF (curve 3) on PMMA vs. the logarithm of surfactant concentration (C). Curves 1, 2 and 3 correspond to the measured values, curves 1′ and 1″ correspond to the values calculated from the exponential function of the second order and Equation (9), respectively; Figure S5: A plot of the contact angle (θ) of aqueous solution of TX165 (curves 1, 1′ and 1″), RL (curve 2) and SF (curve 3 and 3′) on quartz vs. the logarithm of surfactant concentration (C). Curves 1, 2 and 3 correspond to the measured values, curves 1′, and 3′ correspond to the values calculated from the exponential function of the second order and curve 1″ corresponds to the values calculated from Equation (9); Figure S6: A plot of the adhesion tension ($\gamma_{LV}\cos\theta$) vs. the surface tension ($\gamma_{LV}$) of aqueous solutions of TX165, RL and SF for PTFE; Figure S7: A plot of the adhesion tension ($\gamma_{LV}\cos\theta$) vs. the surface tension ($\gamma_{LV}$) of aqueous solutions of TX165 (curve 1), RL (curve 2) and SF (curve 3) for PMMA; Figure S8: A plot of the adhesion tension ($\gamma_{LV}\cos\theta$) vs. the surface tension ($\gamma_{LV}$) of aqueous solutions of TX165 (curve 1), RL (curve 2) and SF (curve 3) for quartz; Figure S9: A plot of the contact angle (θ) of aqueous solution of TX165 + RL mixtures on the PTFE surface vs. the logarithm of the RL concentration (C). Curves 1–16 correspond to the constant TX165 concentration equal to 1 × 10^−8^, 5 × 10^−8^, 1 × 10^−7^, 5 × 10^−7^, 1 × 10^−6^, 5 × 10^−6^, 1 × 10^−5^, 5 × 10^−5^, 1 × 10^−4^, 2 × 10^−4^, 4 × 10^−4^, 6 × 10^−4^, 8 × 10^−4^, 0.001, 0.002 and 0.004 mole/dm^3^, respectively; Figure S10: A plot of the contact angle (θ) of aqueous solution of TX165 + SF mixtures on the PTFE surface vs. the logarithm of the SF concentration (C). Curves 1–16 correspond to the constant TX165 concentration equal to 1 × 10^−8^, 5 × 10^−8^, 1 × 10^−7^, 5 × 10^−7^, 1 × 10^−6^, 5 × 10^−6^, 1 × 10^−5^, 5 × 10^−5^, 1 × 10^−4^, 2 × 10^−4^, 4 × 10^−4^, 6 × 10^−4^, 8 × 10^−4^, 0.001, 0.002 and 0.004 mole/dm^3^, respectively; Figure S11: A plot of the contact angle (θ) of aqueous solution of TX165 + RL on PTFE at the constant RL concentration equal to 5 (a) and 40 mg/dm^3^ (b) vs. the logarithm of TX165 concentration (C). Points 1 correspond to the measured values, curves 2–4 correspond the values calculated form the exponential function of the second order, Equations (13) and (9), respectively; Figure S12: A plot of the contact angle (θ) of aqueous solution of TX165 + RL on PTFE at the constant TX165 concentration equal to 5 × 10^−6^ mole/dm^3^ vs. the logarithm of RL concentration (C). Points 1 correspond to the measured values, curves 2–4 correspond the values calculated form the exponential function of the second order, Equations (13) and (9), respectively; Figure S13: A plot of the contact angle (θ) of aqueous solution of TX165 + SF on PTFE at the constant SF concentration equal to 0.00625 (a), 5 (b) and 40 mg/dm^3^ (c) vs. the logarithm of TX165 concentration (C). Points 1 correspond to the measured values, curves 2–4 correspond the values calculated form the exponential function of the second order, Equations (13) and (9), respectively; Figure S14: A plot of the adhesion tension ($\gamma_{LV}\cos\theta$) vs. the surface tension ($\gamma_{LV}$) of aqueous solutions of TX165 + RL and TX165 + SF mixtures both at the constant biosurfactant and TX165 concentration for PTFE; Figure S15: A plot of the contact angle (θ) of aqueous solution of TX165 + RL mixtures on the PMMA surface vs. the logarithm of RL concentration (C). Curves 1–16 correspond to the constant TX165 concentration equal to 1 × 10^−8^, 5 × 10^−8^, 1 × 10^−7^, 5 × 10^−7^, 1 × 10^−6^, 5 × 10^−6^, 1 × 10^−5^, 5 × 10^−5^, 1 × 10^−4^, 2 × 10^−4^, 4 × 10^−4^, 6 × 10^−4^, 8 × 10^−4^, 0.001, 0.002 and 0.004 mole/dm^3^, respectively; Figure S16: A plot of the contact angle (θ) of aqueous solution of TX165 + SF mixtures on the PMMA surface vs. the logarithm of the SF concentration (C). Curves 1–16 correspond to the constant TX165 concentration equal to 1 × 10^−8^, 5 × 10^−8^, 1 × 10^−7^, 5 × 10^−7^, 1 × 10^−6^, 5 × 10^−6^, 1 × 10^−5^, 5 × 10^−5^, 1 × 10^−4^, 2 × 10^−4^, 4 × 10^−4^, 6 × 10^−4^, 8 × 10^−4^, 0.001, 0.002 and 0.004 mole/dm^3^, respectively; Figure S17: A plot of the contact angle (θ) of aqueous solution of TX165 + RL mixtures on the quartz surface vs. the logarithm of the RL concentration (C). Curves 1–16 correspond to the constant TX165 concentration equal to 1 × 10^−8^, 5 × 10^−8^, 1 × 10^−7^, 5 × 10^−7^, 1 × 10^−6^, 5 × 10^−6^, 1 × 10^−5^, 5 × 10^−5^, 1 × 10^−4^, 2 × 10^−4^, 4 × 10^−4^, 6 × 10^−4^, 8 × 10^−4^, 0.001, 0.002 and 0.004 mole/dm^3^, respectively; Figure S18: A plot of the contact angle (θ) of aqueous solution of TX165 + SF mixtures on the quartz surface vs. the logarithm of the SF concentration (C). Curves 1–16 correspond to the constant TX165 concentration equal to 1 × 10^−8^, 5 × 10^−8^, 1 × 10^−7^, 5 × 10^−7^, 1 × 10^−6^, 5 × 10^−6^, 1 × 10^−5^, 5 × 10^−5^, 1 × 10^−4^, 2 × 10^−4^, 4 × 10^−4^, 6 × 10^−4^, 8 × 10^−4^, 0.001, 0.002 and 0.004 mole/dm^3^, respectively; Figure S19: A plot of the adhesion tension ($\gamma_{LV}\cos\theta$) vs. the surface tension ($\gamma_{LV}$) of aqueous solutions of TX165 + RL at the constant concentration of RL (a) and TX165 (b) at all studied concentration for PMMA; Figure S20: A plot of the adhesion tension ($\gamma_{LV}\cos\theta$) vs. the surface tension ($\gamma_{LV}$) of aqueous solutions of TX165 + SF at the constant concentration of SF (a) and TX165 (b) at all studied concentration for PMMA; Figure S21: A plot of the adhesion tension ($\gamma_{LV}\cos\theta$) vs. the surface tension ($\gamma_{LV}$) of aqueous solutions of TX165 + RL at the constant concentration of RL (a) and TX165 (b) at all studied concentration for quartz; Figure S22: A plot of the adhesion tension ($\gamma_{LV}\cos\theta$) vs. the surface tension ($\gamma_{LV}$) of aqueous solutions of TX165 + SF at the constant concentration of SF (a) and TX165 (b) at all studied concentration for quartz; Figure S23: A plot of the contact angle (θ) for TX165 + RL for PMMA at the constant RL concentration equal to 0.00625 (a), 5 (b) mg/dm^3^ vs. the logarithm of TX165 concentration (C). Points 1 correspond to the measured values, curves 2–4 correspond the values calculated form the exponential function of the second order, Equations (13) and (9), respectively; Figure S24: A plot of the contact angle (θ) for TX165 + SF for PMMA at the constant SF concentration equal to 0.00625 (a), 5 (b) and 40 mg/dm^3^ (c) vs. the logarithm of TX165 concentration (C). Points 1 correspond to the measured values, curves 2–4 correspond the values calculated form the exponential function of the second order, Equations (13) and (9), respectively; Figure S25: A plot of the contact angle (θ) for TX165 + RL for quartz at the constant RL concentration equal to 0.00625 (a), 5 (b) and 40 mg/dm^3^ (c) vs. the logarithm of TX165 concentration (C). Points 1 correspond to the measured values, curves 2–4 correspond the values calculated form the exponential function of the second order, Equations (13) and (9), respectively; Figure S26: A plot of the contact angle (θ) for TX165 + SF for quartz at the constant SF concentration equal to 0.00625 (a), 5 (b) and 40 mg/dm^3^ (c) vs. the logarithm of TX165 concentration (C). Points 1 correspond to the measured values, curves 2–4 correspond the values calculated form the exponential function of the second order, Equations (13) and (9), respectively; Figure S27: A plot of the Gibbs surface excess concentration at the PTFE-water interface ($\Gamma_{SL}$) for TX165 (a, c), RL (b) and SF (d) vs. the logarithm of surfactant concentration (C) calculated from Equations (5) and (15); Figure S28: A plot of the Gibbs surface excess concentration at the PTFE-water interface ($\Gamma_{SL}$) for TX165 (curve 1), RL (2) and their sum (curve 3) vs. the logarithm of surfactant concentration (C) at the constant RL concentration equal to 0.0002 (a), 0.00625 (b), 5 (c) and 40 mg/dm^3^ (d); Figure S29: A plot of the Gibbs surface excess concentration at the PTFE-water interface ($\Gamma_{SL}$) for TX165 (curve 1), RL (2) and their sum (curve 3) vs. the logarithm of surfactant concentration (C) at the constant TX165 concentration equal to 5 × 10^−7^ (a), 1 × 10^−6^ (b), 2 × 10^−4^ (c) and 1 × 10^−3^ mole/dm^3^ (d); Figure S30: A plot of the Gibbs surface excess concentration at the PTFE-water interface ($\Gamma_{SL}$) for TX165 (curve 1), SF (2) and their sum (curve 3) vs. the logarithm of surfactant concentration (C) at the constant SF concentration equal to 0.0002 (a), 0.00625 (b), 5 (c) and 40 mg/dm^3^ (d); Figure S31: A plot of the Gibbs surface excess concentration at the PTFE-water interface ($\Gamma_{SL}$) for TX165 (curve 1), SF (2) and their sum (curve 3) vs. the logarithm of surfactant concentration (C) at the constant TX165 concentration equal to 5 × 10^−7^ (a), 1 × 10^−6^ (b), 2 ⨯10^−4^ (c) and 1 × 10^−3^ mole/dm^3^ (d); Figure S32: A plot of the Gibbs surface excess concentration at the PMMA-water interface ($\Gamma_{SL}$) vs. the logarithm of surfactant concentration (C) for TX165 + RL (a,b) and TX165 +SF (c,d) at the constant TX165 concentration (b, d) and RL (a) as well as SF (c); Figure S33: A plot of the Gibbs surface excess concentration at the PMMA-air interface ($\Gamma_{SV}$) vs. the logarithm of surfactant concentration (C) for TX165 + RL (a, b) and TX165 +SF (c, d) at the constant TX165 concentration (b, d) and RL (a) as well as SF (c); Figure S34: A plot of the Gibbs surface excess concentration at the quartz-water interface ($\Gamma_{SL}$) vs. the logarithm of surfactant concentration (C) for TX165 + RL (a,b) and TX165 +SF (c,d) at the constant TX165 concentration (b,d) and RL (a) as well as SF (c); Figure S35: A plot of the Gibbs surface excess concentration at the quartz-air interface ($\Gamma_{SV}$) vs. the logarithm of surfactant concentration (C) for TX165 + RL (a, b) and TX165 +SF (c, d) at the constant TX165 concentration (b,d) and RL (a) as well as SF (c); Figure S36: The difference between the Gibbs surface excess concentration at the PMMA-water and PMMA-air interface vs. the logarithm of surfactant concentration (C) for TX165 + RL (a) and TX165 +SF (b). Curves 1–4 correspond to the constant biosurfactant concentration equal to 0.0002, 0.00625, 5 and 40 mg/dm^3^; Figure S37: A plot of the difference between the Gibbs surface excess concentration at the quartz-water and quartz-air interface vs. the logarithm of surfactant concentration (C) for TX165 + RL (a) and TX165 +SF (b). Curves 1–4 correspond to the constant biosurfactant concentration equal to 0.0002, 0.00625, 5 and 40 mg/dm^3^; Figure S38: A plot of the difference between the values of the PMMA(quartz)-air interface tension ($\Delta \gamma_{SV}$) at the TX165 + biosurfactant mixture concentration equal to zero and equal to a given value (curve 1) and between the PMMA(quartz)-water (curve 2) and PMMA(quartz)-solution (curve 2) ($\Delta \gamma_{SL}$) as well as the difference between the values of contact angle of the water and solution on the PMMA (a,b) and quartz (c,d) ($\Delta \gamma_{\theta }$) (curve 3) at the constant RL and SF concentration equal to 0.00625 mg/dm^3^ vs. the logarithm of surfactant concentration (C).
