# Supplementary material for: Wetting Properties of Rhamnolipid and Surfactin Mixtures with Triton X-165
Source: Molecules. 2022 Jul 23;27(15):4706. doi: 10.3390/molecules27154706 (PMC9330438; doi:10.3390/molecules27154706)
Supplement: Supplementary file 1 [file molecules-27-04706-s001.zip › molecules-1809054-supplementary.pdf]

Supplementary Materials

Wetting properties of rhamnolipid and surfactin mixtures with Triton X-165

Edyta Rekiel [1], Anna Zdziennicka [1], Katarzyna Szymczyk [1] and Bronisław Jańczuk [1,*]

Department of Interfacial Phenomena, Institute of Chemical Sciences, Faculty of Chemistry, Maria Curie-Skłodowska University in Lublin. Maria Curie-Skłodowska Sq.3, 20-031 Lublin, Poland

*Correspondence: bronislaw.janczuk@poczta.umcs.lublin.pl ; Tel.: (48-81) 537-56-70

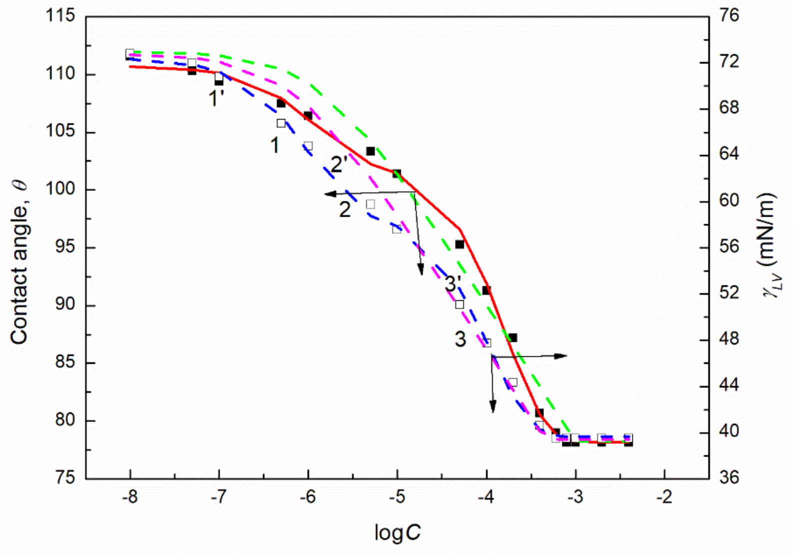

Figure S1. A plot of the TX165 aqueous solution contact angle (θ) surface tension (γ_{LV}) (curves 1 - 3) and contact angle (θ) on PTFE (curves 1′ - 3′) vs. the logarithm of TX165 concentration (C). Curves 1 and 1′ correspond to the measured values, curves 2 and 2′ to values calculated from the exponential function of the second order, curves 3 and 3′ correspond to the values calculated from Eqs. (9) and (11), respectively.

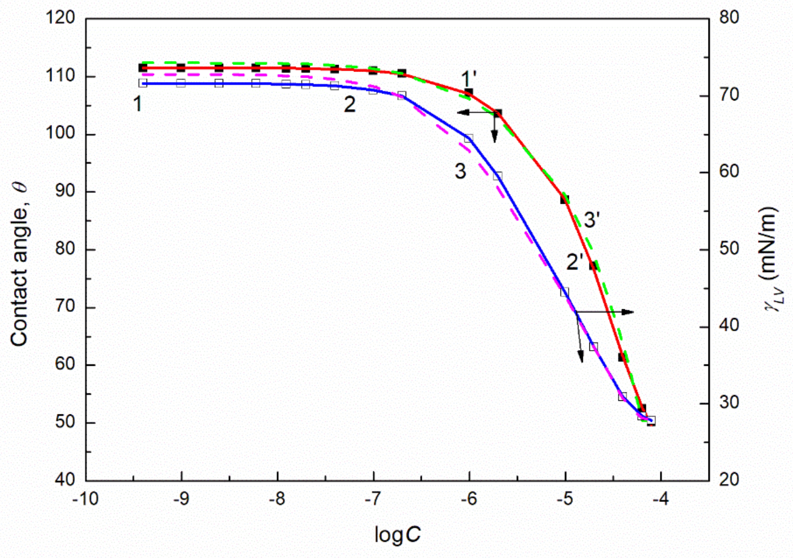

Figure S2. A plot of the RL aqueous solution surface tension (γ_{LV}) (curves 1 - 3) and contact angle (θ) on PTFE (curves 1′ - 3′) vs. the logarithm of RL concentration (C). Curves 1 and 1′ correspond to the measured values, curves 2 and 2′ to values calculated from the exponential function of the second order, curves 3 and 3′ correspond to the values calculated from Eqs. (9) and (11), respectively.

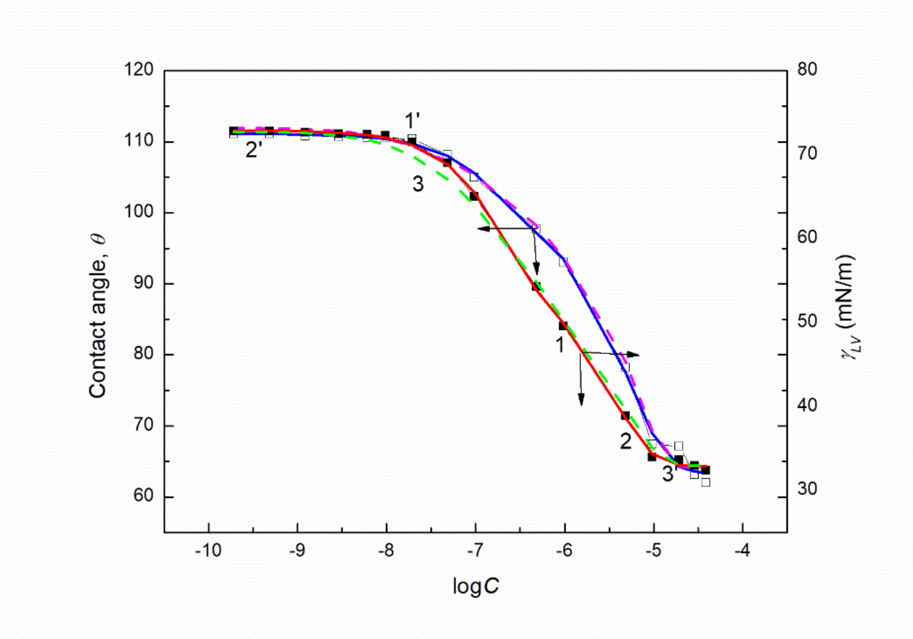

Figure S3. A plot of the SF aqueous solution surface tension (γ_{LV}) (curves 1 - 3) and contact angle (θ) on PTFE (curves 1′ - 3′) vs. the logarithm of SF concentration (C). Curves 1 and 1′ correspond the measured values, curves 2 and 2′ to values calculated from the exponential function of the second order, curves 3 and 3′ correspond to the values calculated from Eqs. (9) and (11), respectively.

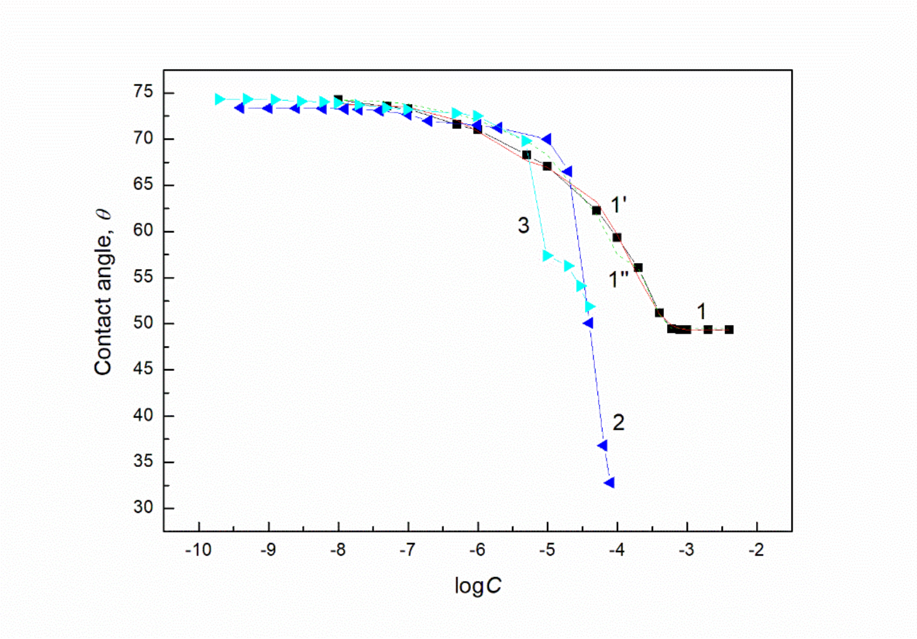

Figure S4. A plot of the contact angle (θ) of aqueous solution of TX165 (curves 1, 1′ and 1″), RL (curve 2) and SF (curve 3) on PMMA vs. the logarithm of surfactant concentration (C). Curves 1, 2 and 3 correspond to the measured values, curves 1′ and 1″ correspond to the values calculated from the exponential function of the second order and Eq. (9), respectively.

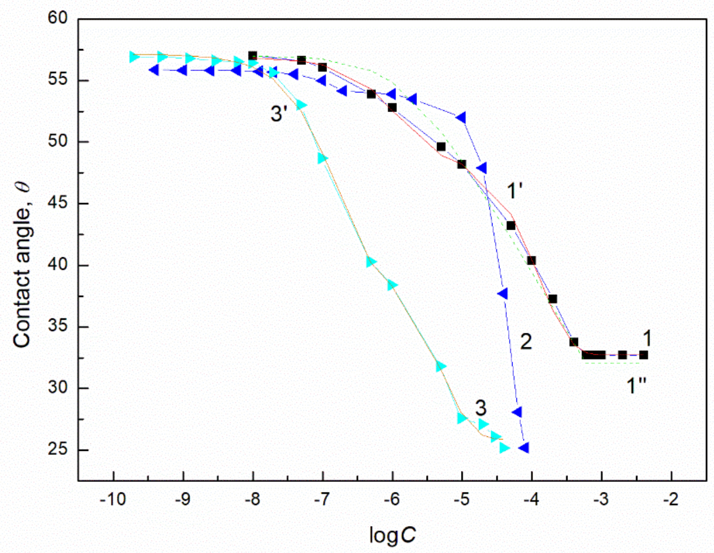

Figure S5. A plot of the contact angle (θ) of aqueous solution of TX165 (curves 1, 1′ and 1″), RL (curve 2) and SF (curve 3 and 3′) on quartz vs. the logarithm of surfactant concentration (C). Curves 1, 2 and 3 correspond to the measured values, curves 1′, and 3′ correspond to the values calculated from the exponential function of the second order and curve 1″ corresponds to the values calculated from Eq. (9).

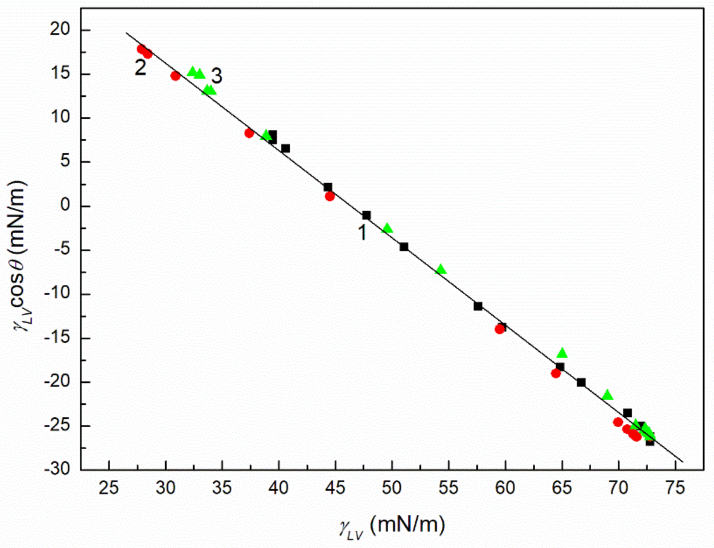

Figure S6. A plot of the adhesion tension ($\gamma_{LV}\cos\theta$) vs. the surface tension (γ_{LV}) of aqueous solutions of TX165 (points 1), RL (points 2) and SF (points 3) for PTFE.

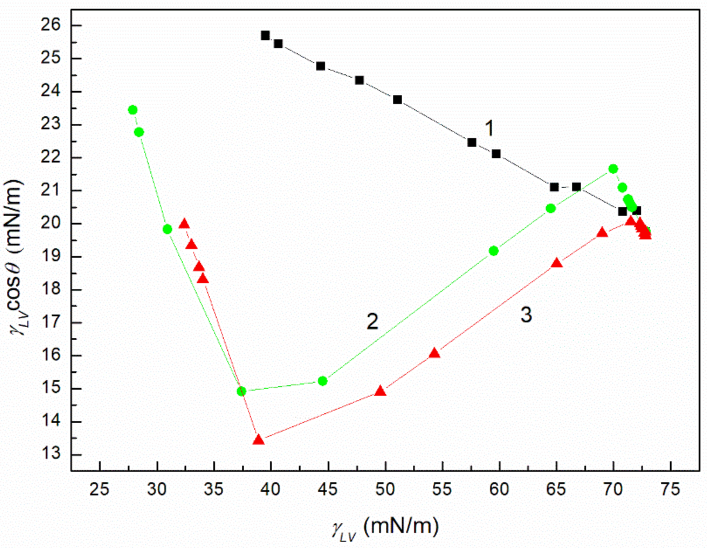

Figure S7. A plot of the adhesion tension ($\gamma_{LV}\cos\theta$) vs. the surface tension (γ_{LV}) of aqueous solutions of TX165 (curve 1), RL (curve 2) and SF (curve 3) for PMMA.

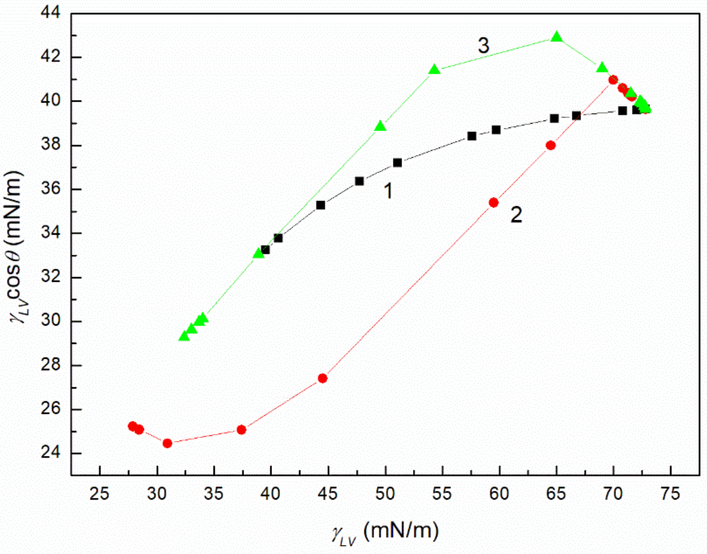

Figure S8. A plot of the adhesion tension ($\gamma_{LV}\cos\theta$) vs. the surface tension (γ_{LV}) of aqueous solutions of TX165 (curve 1), RL (curve 2) and SF (curve 3) for quartz.

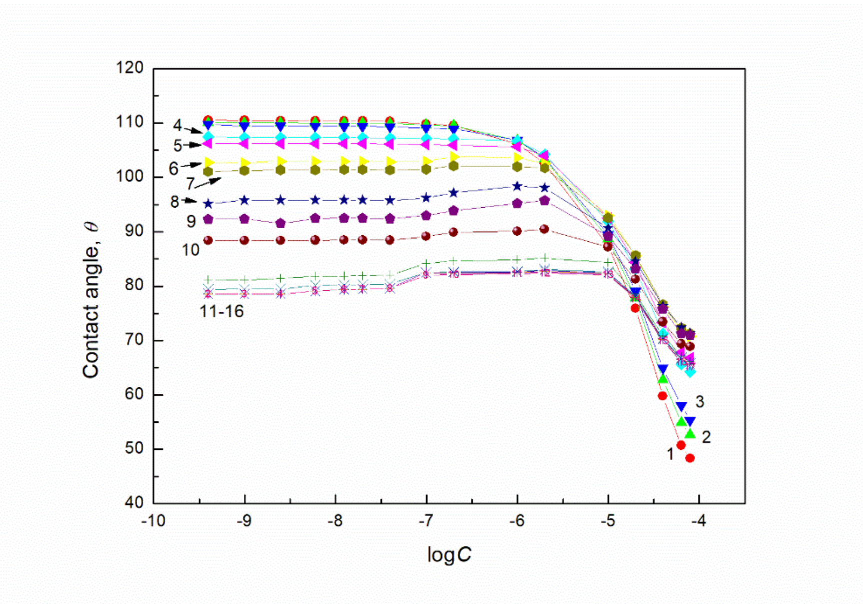

Figure S9. A plot of the contact angle (θ) of aqueous solution of TX165 + RL mixtures on the PTFE surface vs. the logarithm of the RL concentration (C). Curves 1 – 16 correspond to the constant TX165 concentration equal to 1 × 10^{-8}, 5 × 10^{-8}, 1 × 10^{-7}, 5 × 10^{-7}, 1 × 10^{-6}, 5 × 10^{-6}, 1 × 10^{-5}, 5 × 10^{-5}, 1 × 10^{-4}, 2 × 10^{-4}, 4 × 10^{-4}, 6 × 10^{-4}, 8 × 10^{-4}, 0.001, 0.002 and 0.004 mole/dm^3, respectively.

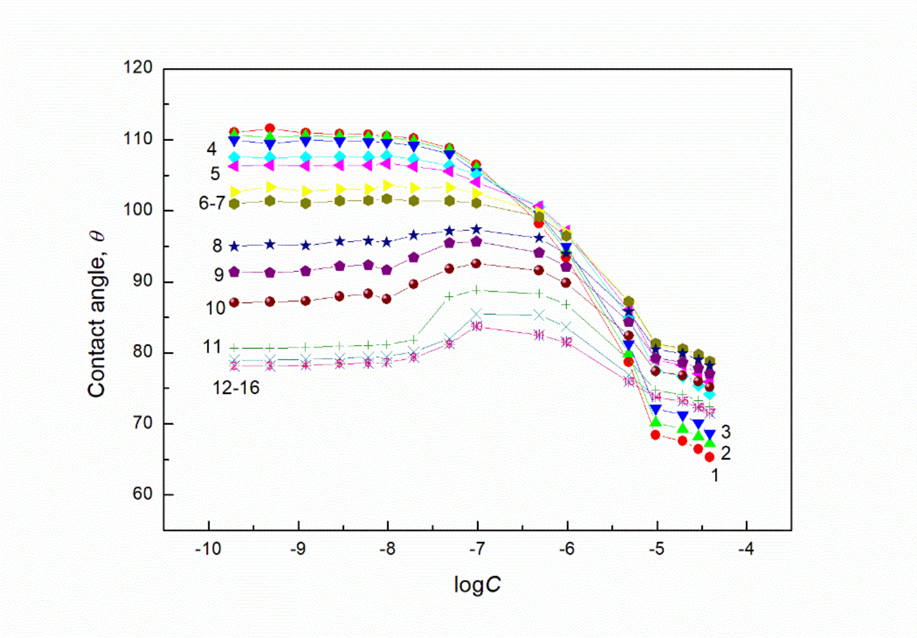

Figure S10. A plot of the contact angle (θ) of aqueous solution of TX165 + SF mixtures on the PTFE surface vs. the logarithm of the SF concentration (C). Curves 1 – 16 correspond to the constant TX165 concentration equal to 1 × 10^{-8}, 5 × 10^{-8}, 1 × 10^{-7}, 5 × 10^{-7}, 1 × 10^{-6}, 5 × 10^{-6}, 1 × 10^{-5}, 5 × 10^{-5}, 1 × 10^{-4}, 2 × 10^{-4}, 4 × 10^{-4}, 6 × 10^{-4}, 8 × 10^{-4}, 0.001, 0.002 and 0.004 mole/dm^3, respectively.

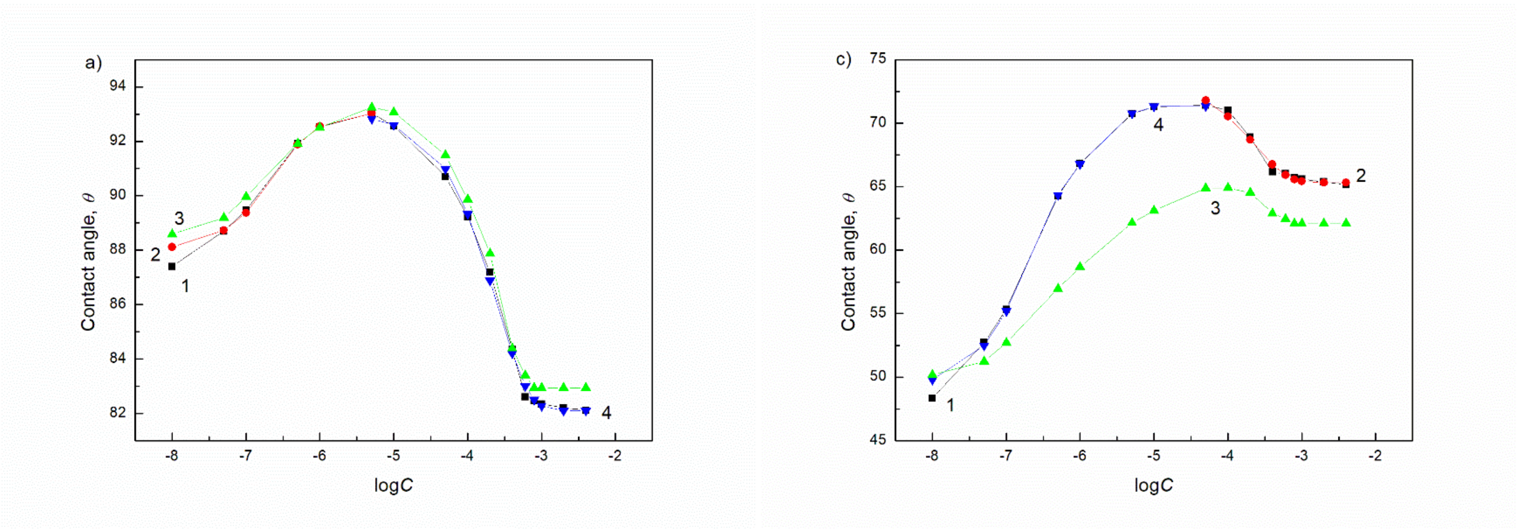

Figure S11. A plot of the contact angle (θ) of aqueous solution of TX165 + RL on PTFE at the constant RL concentration equal to 5 (a) and 40 mg/dm^3 (c) vs. the logarithm of TX165 concentration (C). Points 1 correspond to the measured values, curves 2 – 4 correspond the values calculated form the exponential function of the second order, Eqs. (13) and (9), respectively.

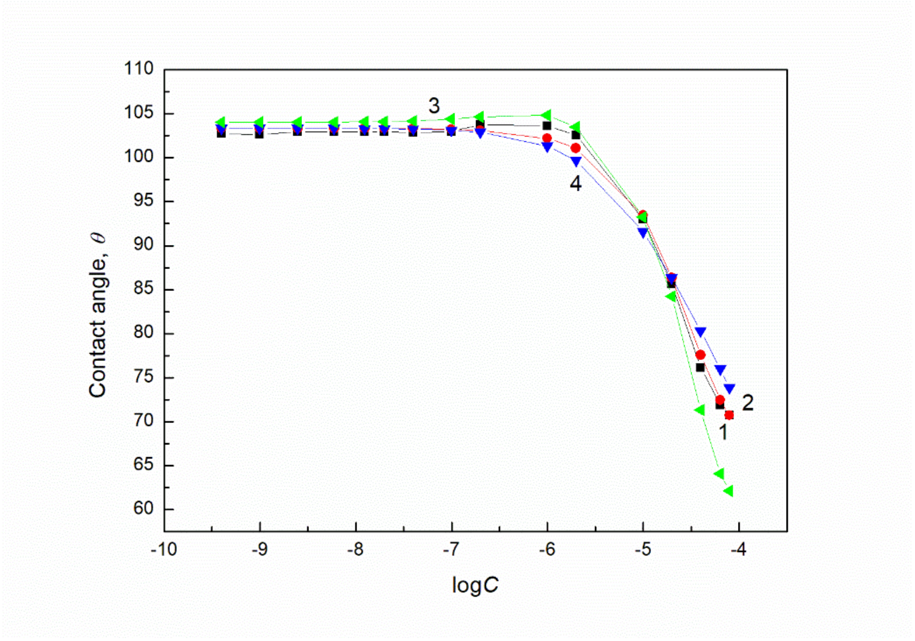

Figure S12. A plot of the contact angle (θ) of aqueous solution of TX165 + RL on PTFE at the constant TX165 concentration equal to 5 x 10^{-6} mole/dm^3 vs. the logarithm of RL concentration (C). Points 1 correspond to the measured values, curves 2 – 4 correspond the values calculated form the exponential function of the second order, Eqs. (13) and (9), respectively.

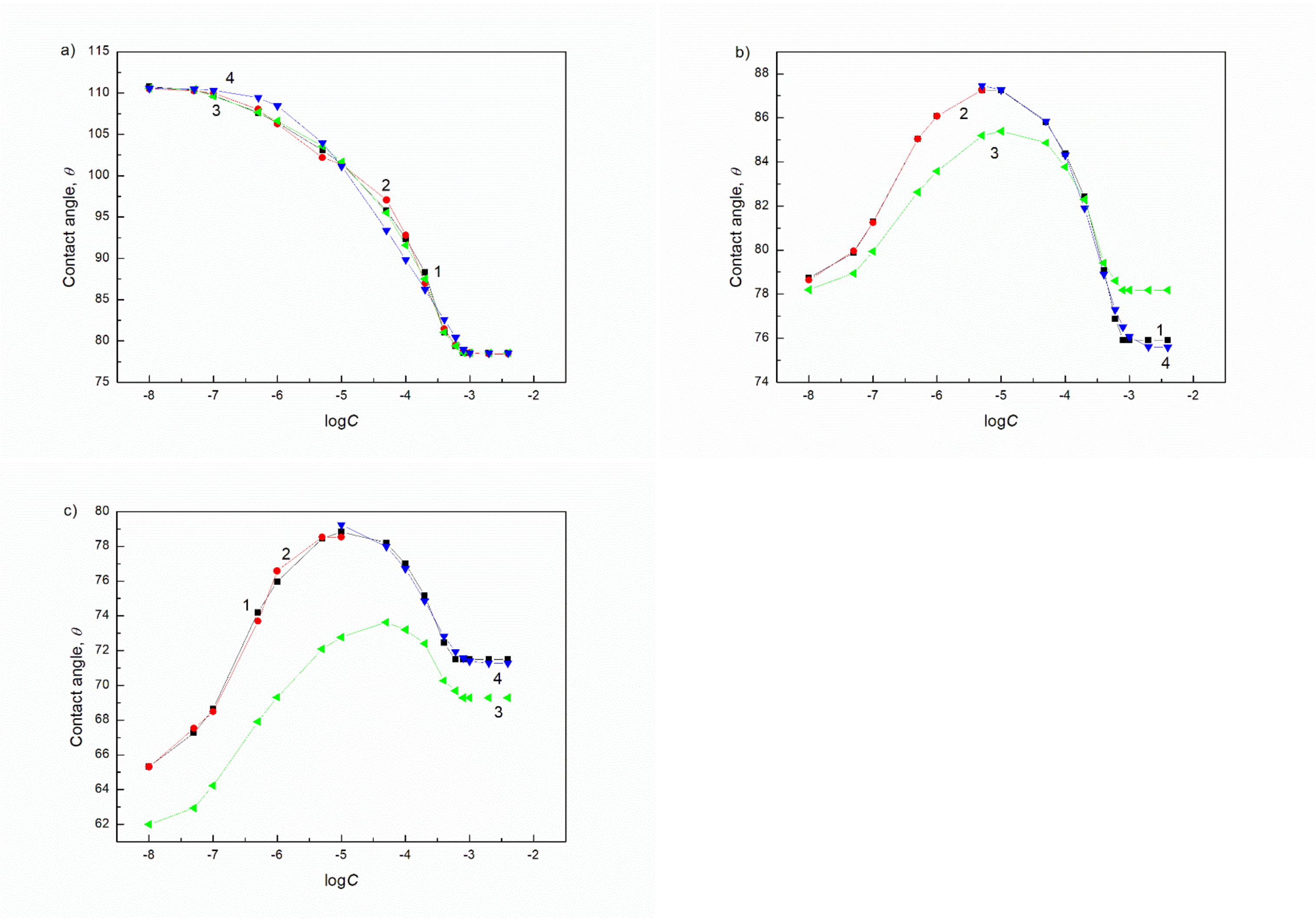

Figure S13. A plot of the contact angle (θ) of aqueous solution of TX165 + SF on PTFE at the constant SF concentration equal to 0.00625 (a), 5 (b) and 40 mg/dm^3 (c) vs. the logarithm of TX165 concentration (C). Points 1 correspond to the measured values, curves 2 – 4 correspond the values calculated form the exponential function of the second order, Eqs. (13) and (9), respectively.

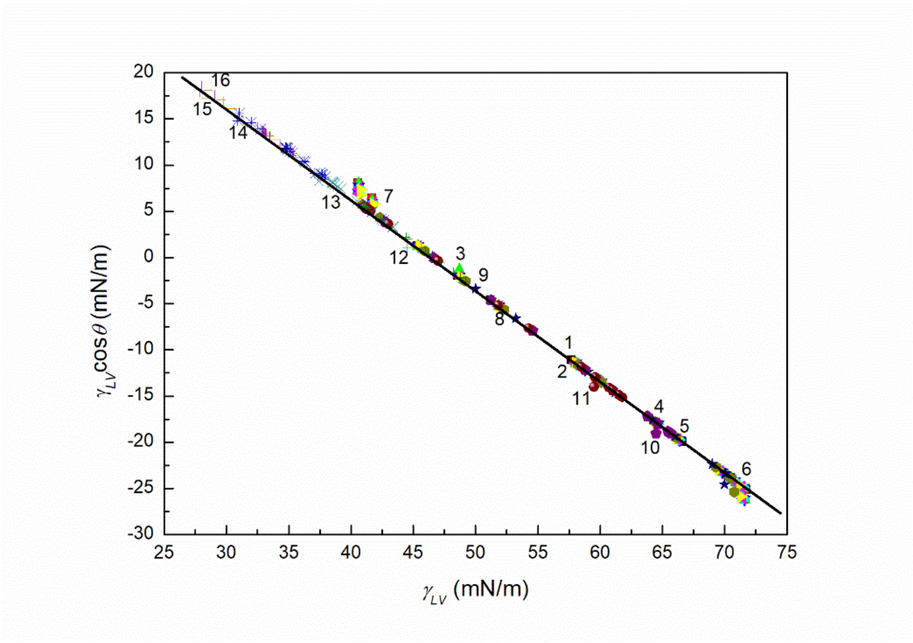

Figure S14. A plot of the adhesion tension ($\gamma_{LV}\cos\theta$) vs. the surface tension (γ_{LV}) of aqueous solutions of TX165 + RL and TX165 + SF mixtures both at the constant biosurfactant and TX165 concentration for PTFE.

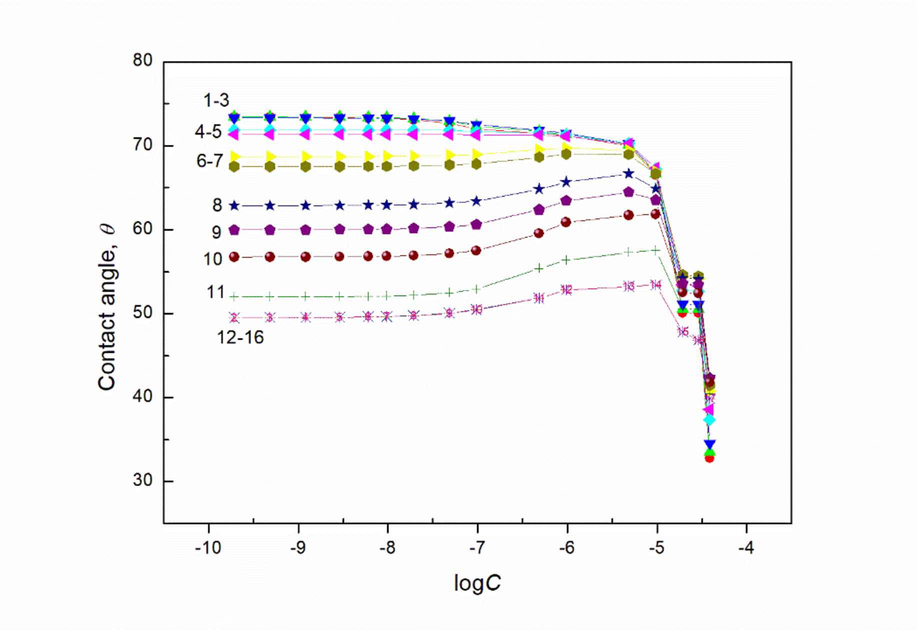

Figure S15. A plot of the contact angle (θ) of aqueous solution of TX165 + RL mixtures on the PMMA surface vs. the logarithm of RL concentration (C). Curves 1 – 16 correspond to the constant TX165 concentration equal to 1 × 10^{-8}, 5 × 10^{-8}, 1 × 10^{-7}, 5 × 10^{-7}, 1 × 10^{-6}, 5 × 10^{-6}, 1 × 10^{-5}, 5 × 10^{-5}, 1 × 10^{-4}, 2 × 10^{-4}, 4 × 10^{-4}, 6 × 10^{-4}, 8 × 10^{-4}, 0.001, 0.002 and 0.004 mole/dm^3, respectively.

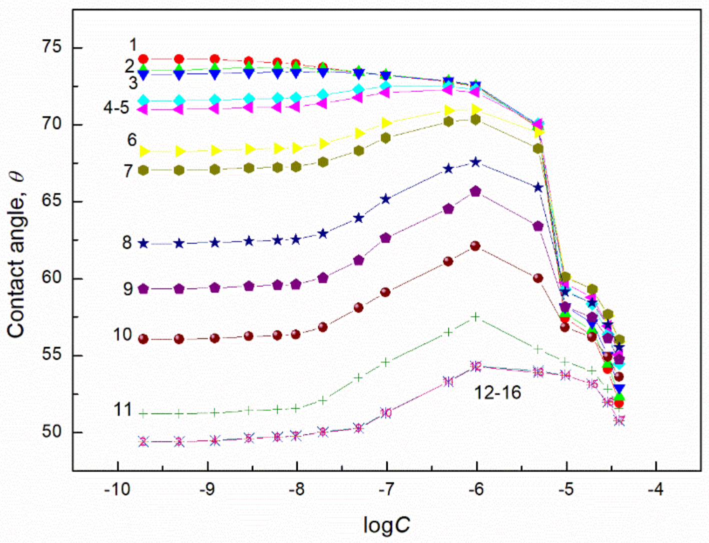

Figure S16. A plot of the contact angle (θ) of aqueous solution of TX165 + SF mixtures on the PMMA surface vs. the logarithm of the SF concentration (C). Curves 1 – 16 correspond to the constant TX165 concentration equal to 1×10^{-8}, 5×10^{-8}, 1×10^{-7}, 5×10^{-7}, 1×10^{-6}, 5×10^{-6}, 1×10^{-5}, 5×10^{-5}, 1×10^{-4}, 2×10^{-4}, 4×10^{-4}, 6×10^{-4}, 8×10^{-4}, 0.001, 0.002 and 0.004 mole/dm^3, respectively.

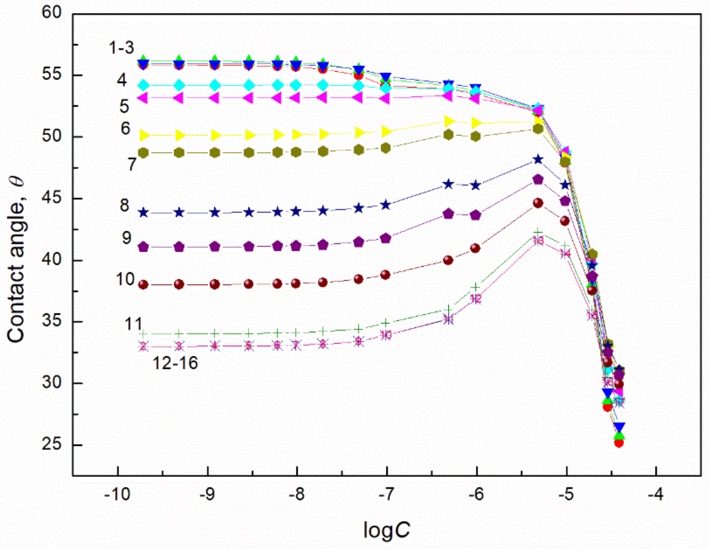

Figure S17. A plot of the contact angle (θ) of aqueous solution of TX165 + RL mixtures on the quartz surface vs. the logarithm of the RL concentration (C). Curves 1 – 16 correspond to the constant TX165 concentration equal to 1×10^{-8}, 5×10^{-8}, 1×10^{-7}, 5×10^{-7}, 1×10^{-6}, 5×10^{-6}, 1×10^{-5}, 5×10^{-5}, 1×10^{-4}, 2×10^{-4}, 4×10^{-4}, 6×10^{-4}, 8×10^{-4}, 0.001, 0.002 and 0.004 mole/dm^3, respectively.

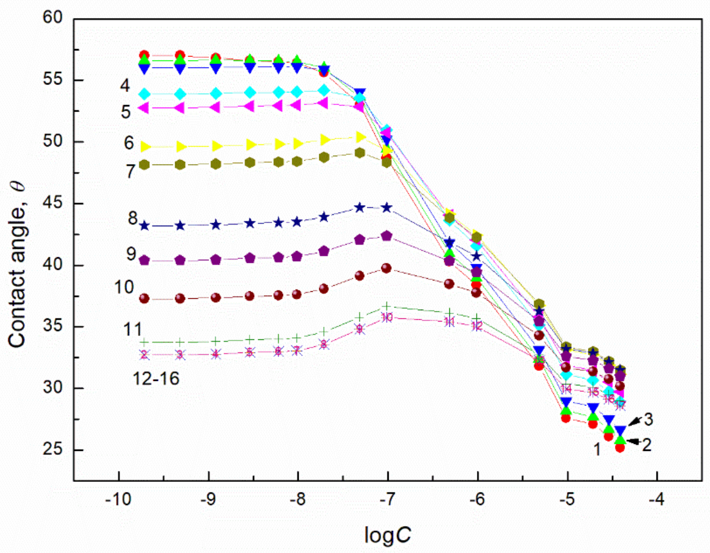

Figure S18. A plot of the contact angle (θ) of aqueous solution of TX165 + SF mixtures on the quartz surface vs. the logarithm of the SF concentration (C). Curves 1 – 16 correspond to the constant TX165 concentration equal to 1×10^{-8}, 5×10^{-8}, 1×10^{-7}, 5×10^{-7}, 1×10^{-6}, 5×10^{-6}, 1×10^{-5}, 5×10^{-5}, 1×10^{-4}, 2×10^{-4}, 4×10^{-4}, 6×10^{-4}, 8×10^{-4}, 0.001, 0.002 and 0.004 mole/dm^3, respectively.

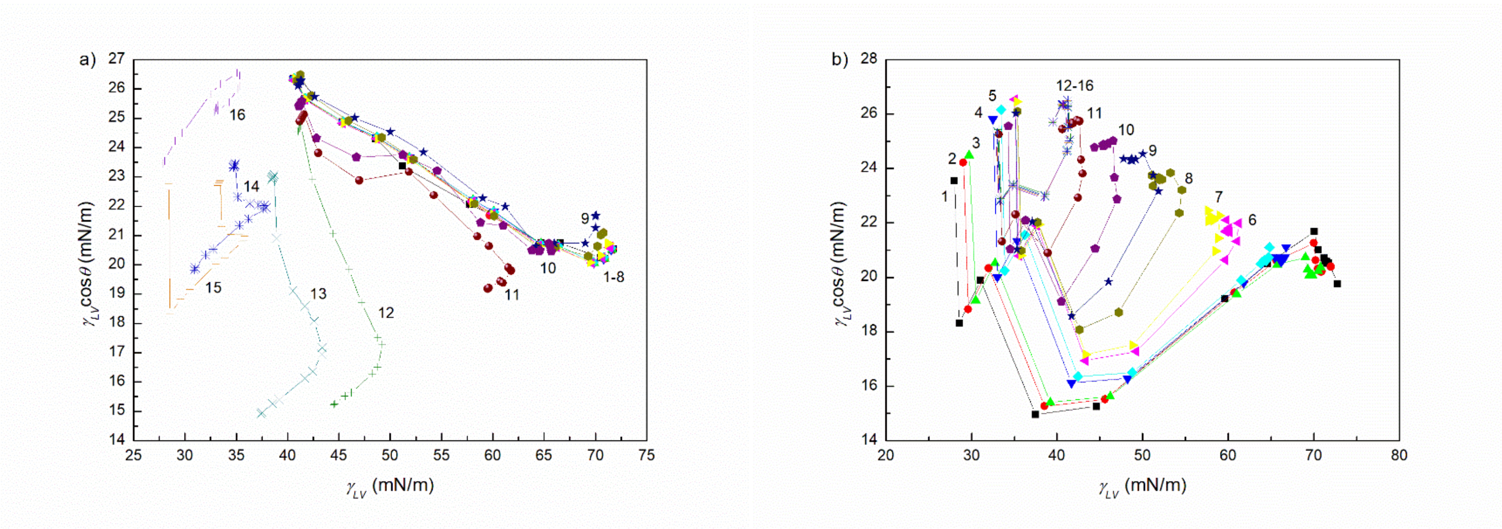

Figure S19. A plot of the adhesion tension ($\gamma_{LV}\cos\theta$) vs. the surface tension (γ_{LV}) of aqueous solutions of TX165 + RL at the constant concentration of RL (a) and TX165 (b) at all studied concentration for PMMA.

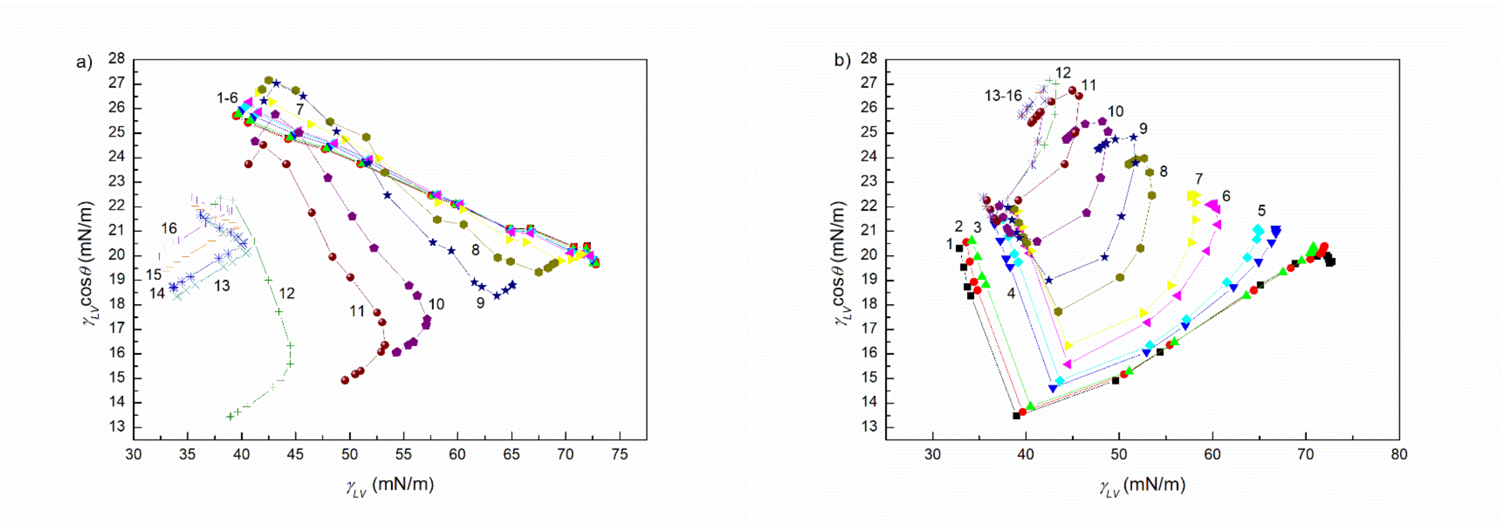

Figure S20. A plot of the adhesion tension ($\gamma_{LV}\cos\theta$) vs. the surface tension (γ_{LV}) of aqueous solutions of TX165 + SF at the constant concentration of SF (a) and TX165 (b) at all studied concentration for PMMA.

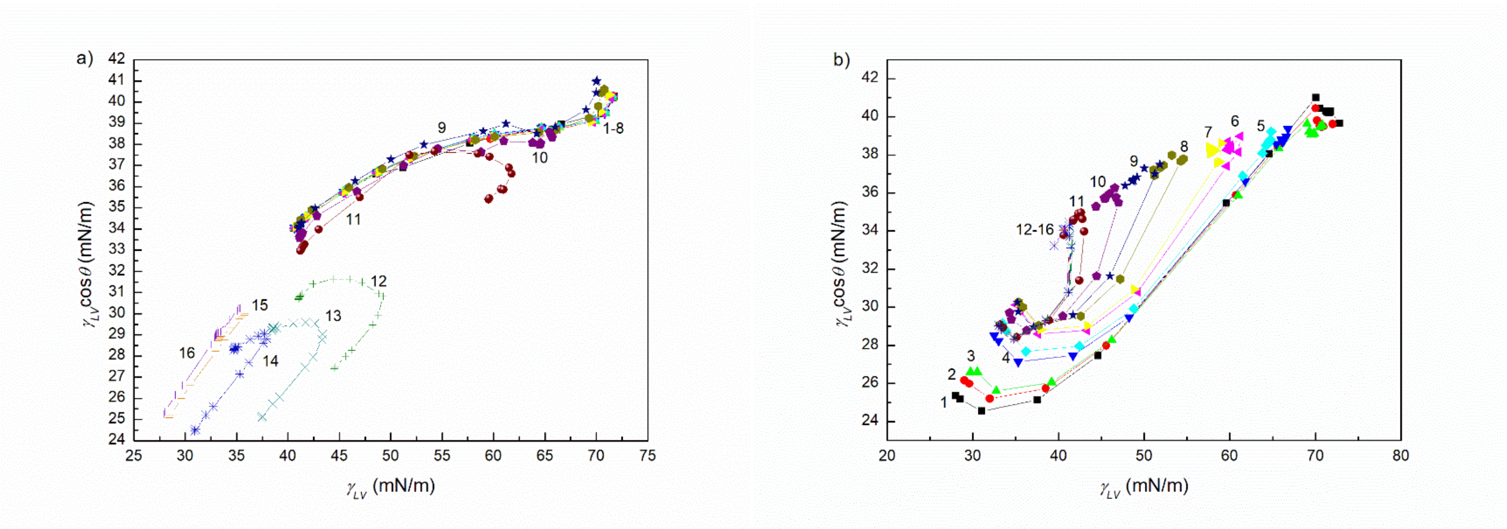

Figure S21. A plot of the adhesion tension ($\gamma_{LV}\cos\theta$) vs. the surface tension (γ_{LV}) of aqueous solutions of TX165 + RL at the constant concentration of RL (a) and TX165 (b) at all studied concentration for quartz.

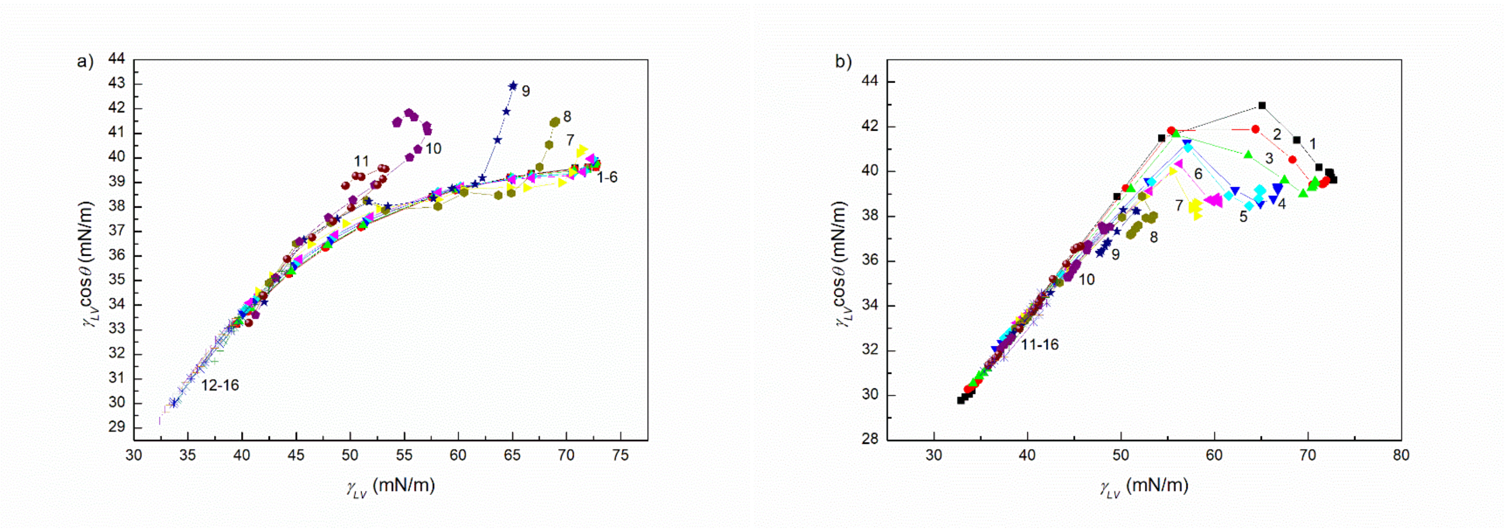

Figure S22. A plot of the adhesion tension ($\gamma_{LV}\cos\theta$) vs. the surface tension (γ_{LV}) of aqueous solutions of TX165 + SF at the constant concentration of SF (a) and TX165 (b) at all studied concentration for quartz.

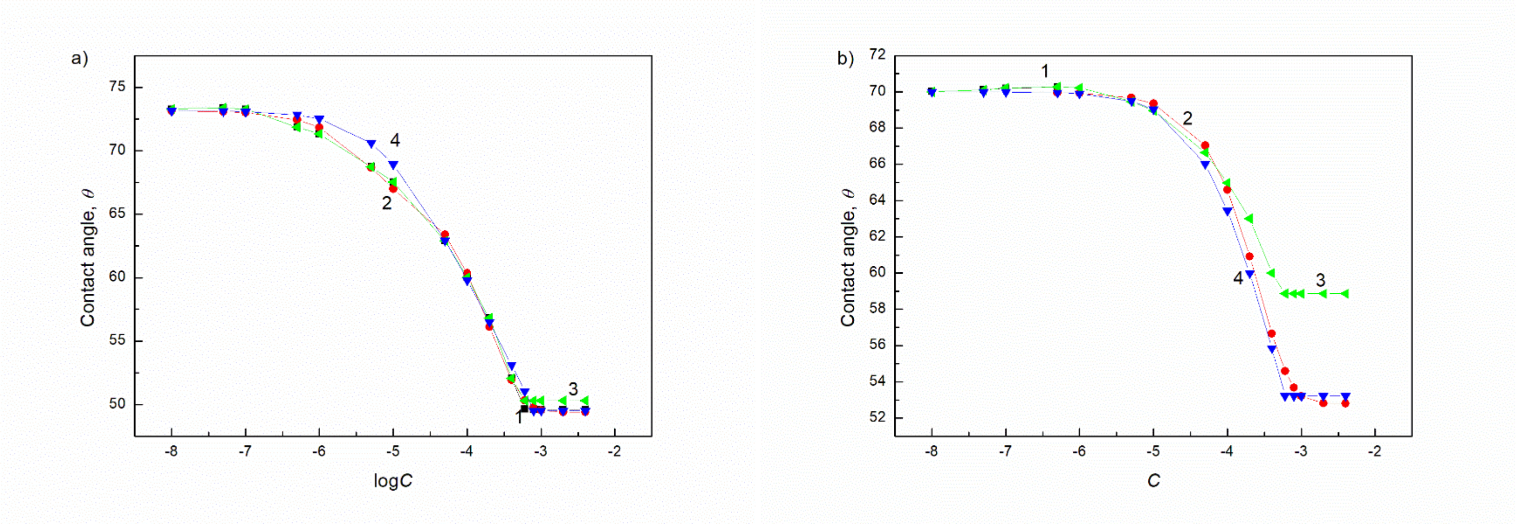

Figure S23. A plot of the contact angle (θ) for TX165 + RL for PMMA at the constant RL concentration equal to 0.00625 (a) and 5 (b) vs. the logarithm of TX165 concentration (C). Points 1 correspond to the measured values, curves 2 – 4 correspond the values calculated from the exponential function of the second order, Eqs. (13) and (9), respectively.

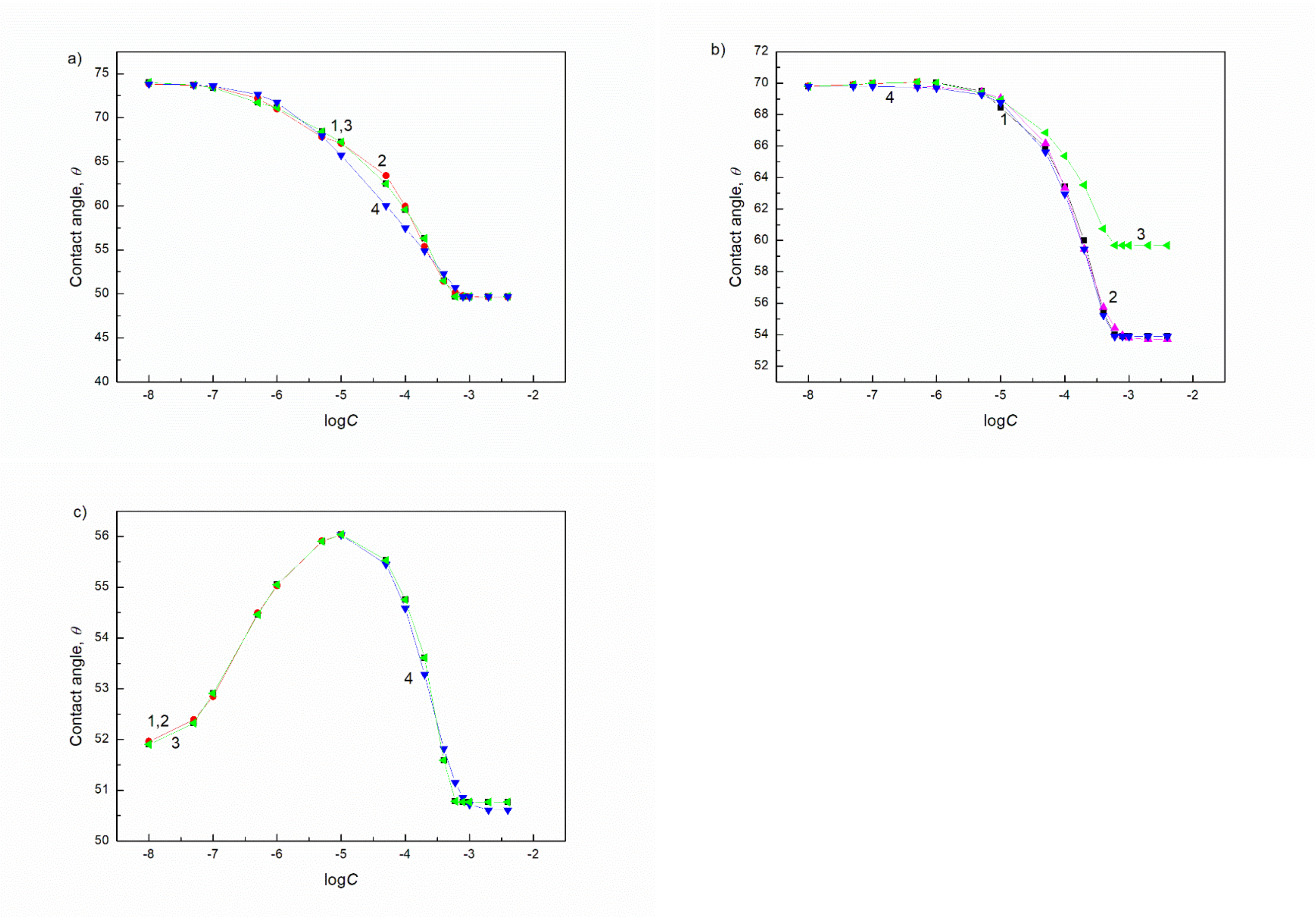

Figure S24. A plot of the contact angle (θ) for TX165 + SF for PMMA at the constant SF concentration equal to 0.00625 (a), 5 (b) and 40 mg/dm^3 (c) vs. the logarithm of TX165 concentration (C). Points 1 correspond to the measured values, curves 2 – 4 correspond the values calculated form the exponential function of the second order, Eqs. (13) and (9), respectively.

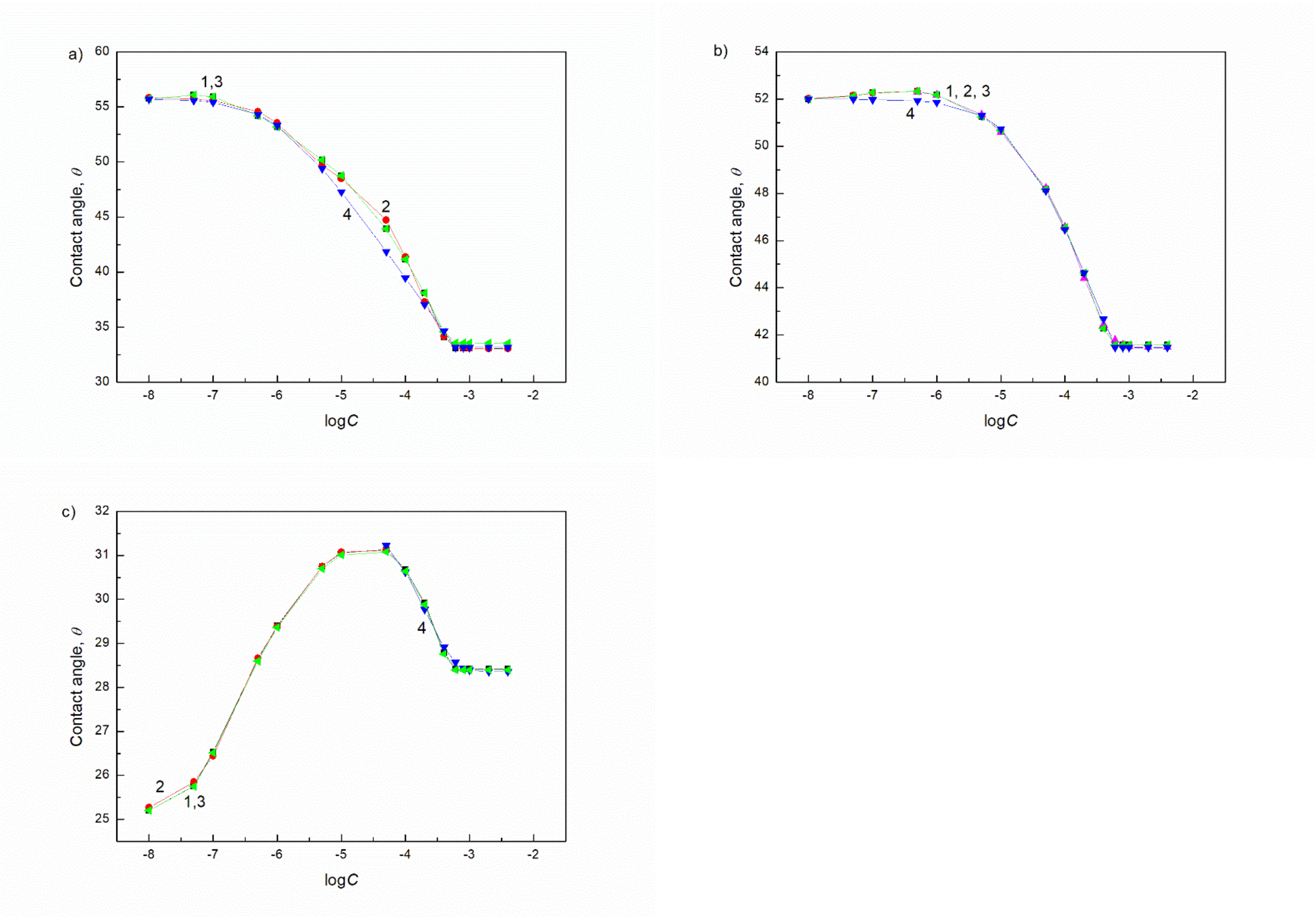

Figure S25. A plot of the contact angle (θ) for TX165 + RL for quartz at the constant RL concentration equal to 0.00625 (a), 5 (b) and 40 mg/dm^3 (c) vs. the logarithm of TX165 concentration (C). Points 1 correspond to the measured values, curves 2 – 4 correspond the values calculated form the exponential function of the second order, Eqs. (13) and (9), respectively.

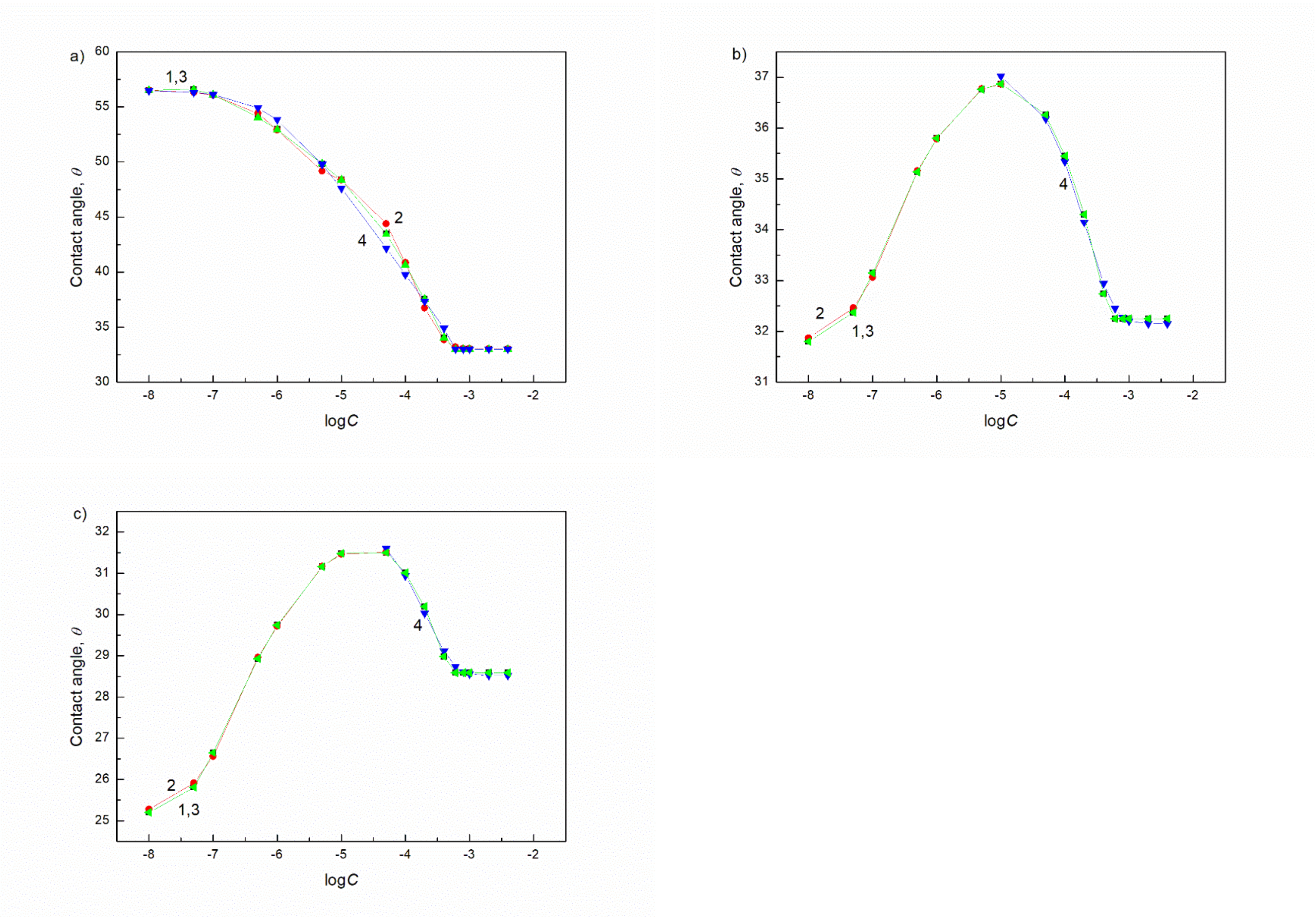

Figure S26. A plot of the contact angle (θ) for TX165 + SF for quartz at the constant SF concentration equal to 0.00625 (a), 5 (b) and 40 mg/dm^3 (c) vs. the logarithm of TX165 concentration (C). Points 1 correspond to the measured values, curves 2 – 4 correspond the values calculated form the exponential function of the second order, Eqs. (13) and (9), respectively.

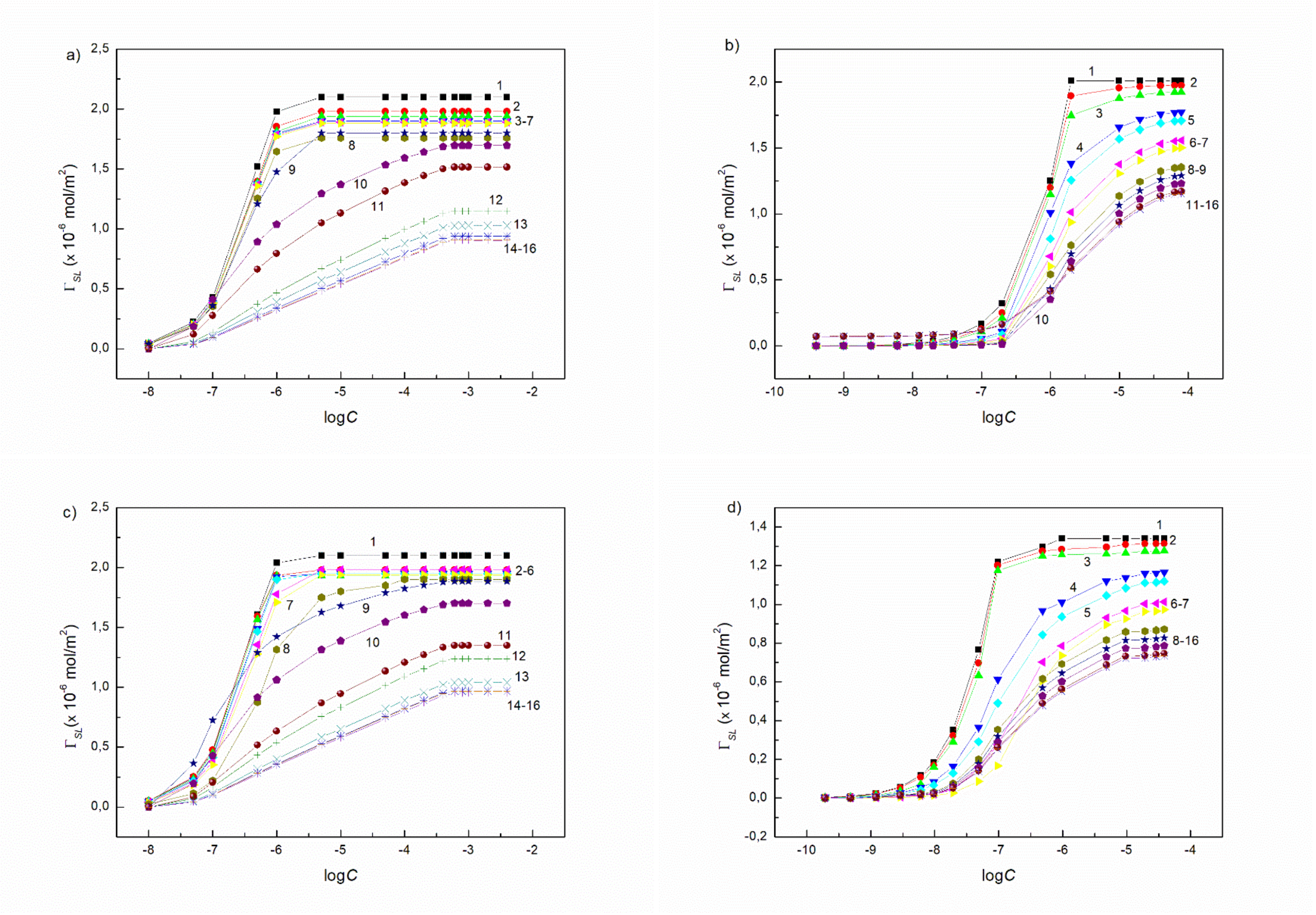

Figure S27. A plot of the Gibbs surface excess concentration at the PTFE-water interface (Γ_{SL}) for TX165 (a, c), RL (b) and SF (d) vs. the logarithm of surfactant concentration (C) calculated from Eqs. (5) and (15).

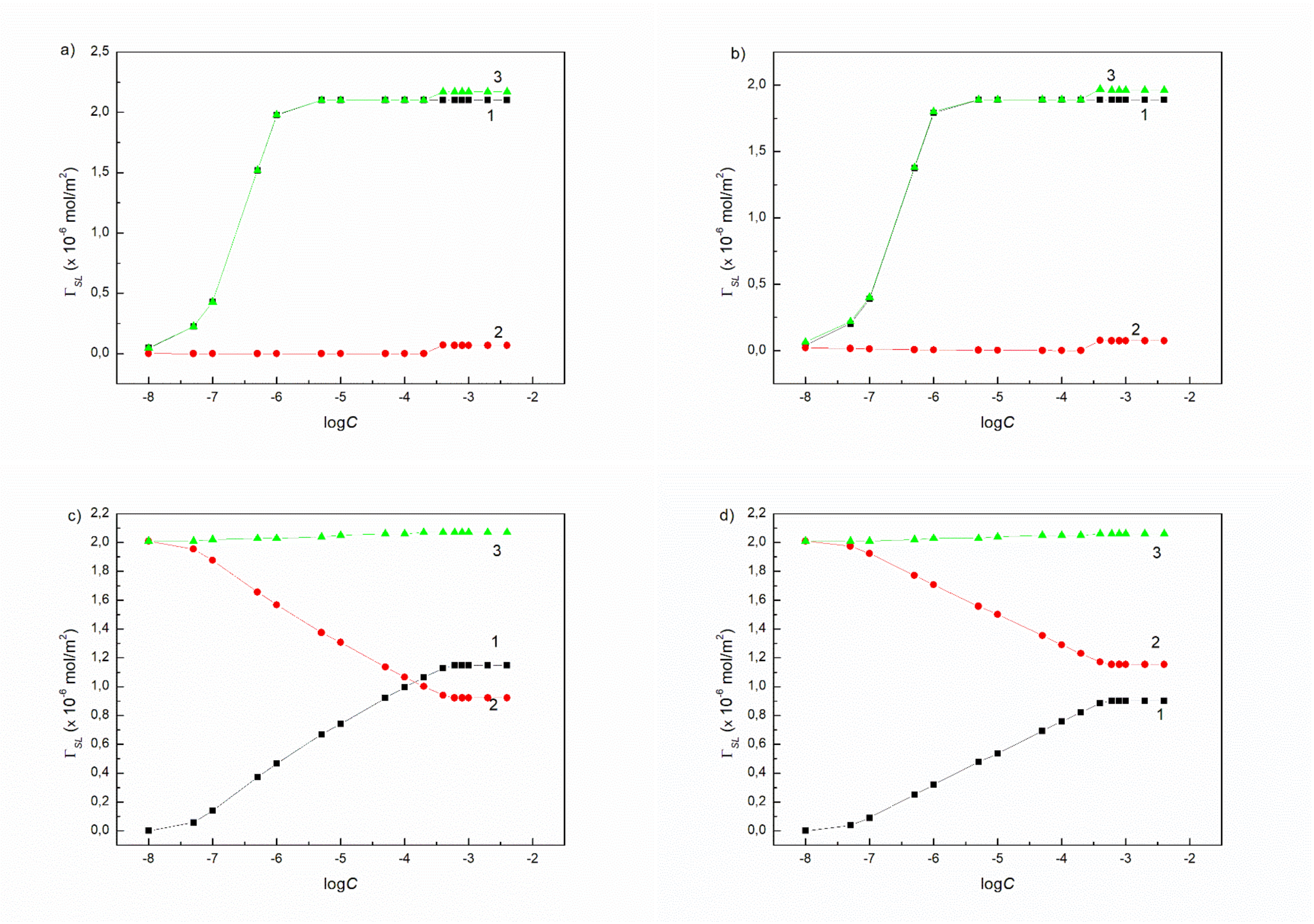

Figure S28. A plot of the Gibbs surface excess concentration at the PTFE-water interface (Γ_{SL}) for TX165 (curve 1), RL (2) and their sum (curve 3) vs. the logarithm of surfactant concentration (C) at the constant RL concentration equal to 0.0002 (a), 0.00625 (b), 5 (c) and 40 mg/dm^3 (d).

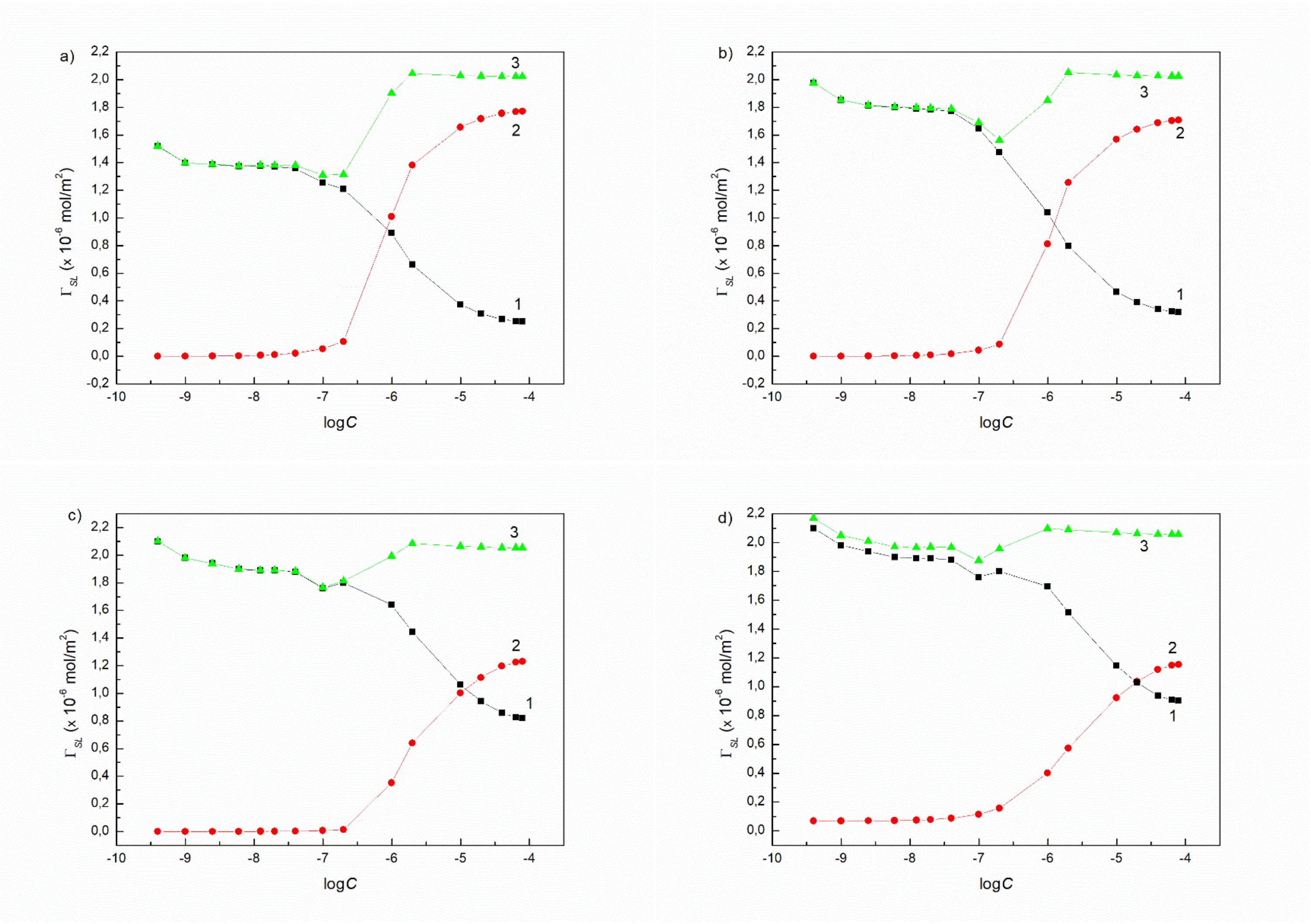

Figure S29. A plot of the Gibbs surface excess concentration at the PTFE-water interface (Γ_{SL}) for TX165 (curve 1), RL (2) and their sum (curve 3) vs. the logarithm of surfactant concentration (C) at the constant TX165 concentration equal to 5 × 10^{-7} (a), 1 × 10^{-6} (b), 2 × 10^{-4} (c) and 1 × 10^{-3} mole/dm^3 (d).

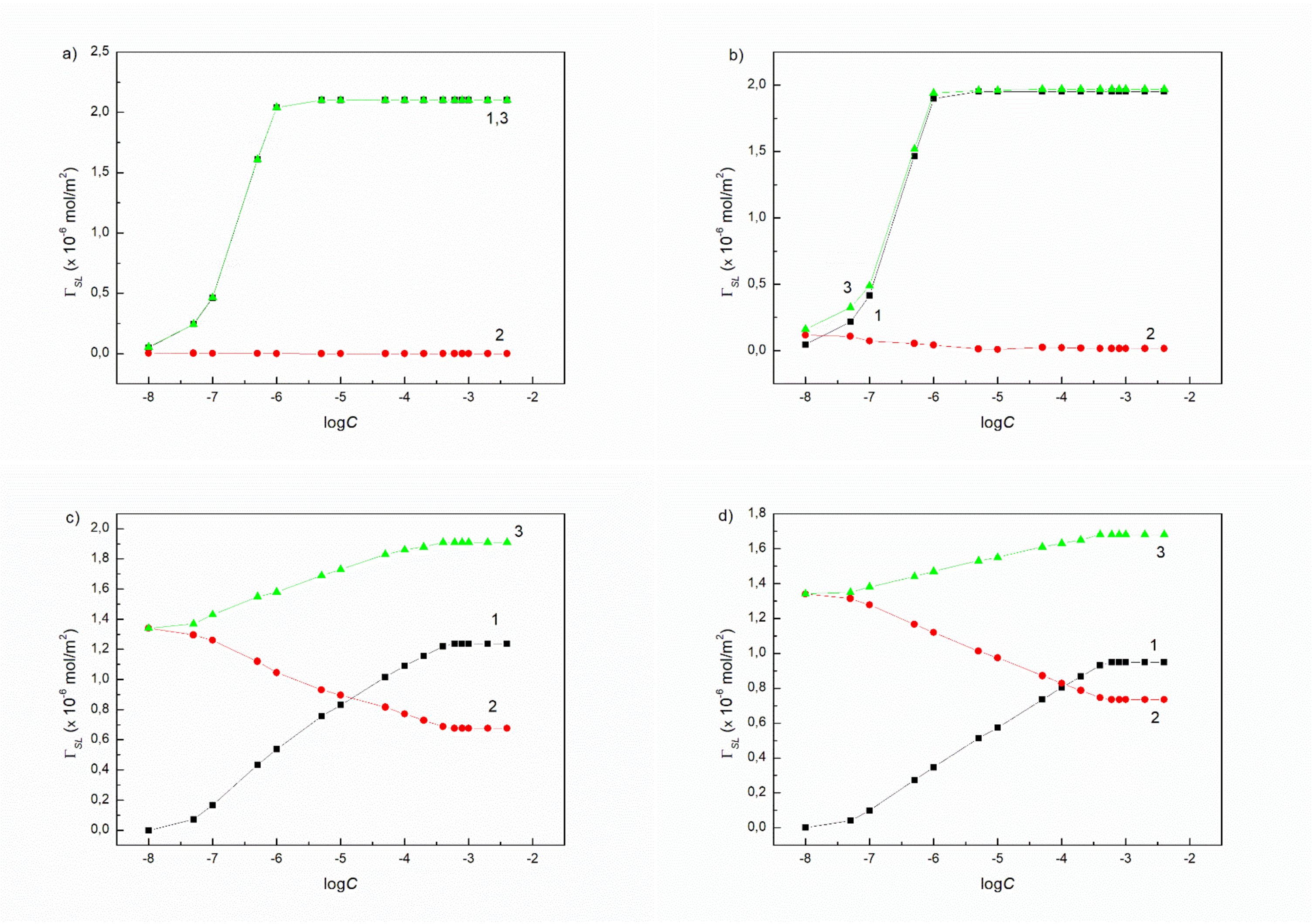

Figure S30. A plot of the Gibbs surface excess concentration at the PTFE-water interface (Γ_{SL}) for TX165 (curve 1), SF (2) and their sum (curve 3) vs. the logarithm of surfactant concentration (C) at the constant SF concentration equal to 0.0002 (a), 0.00625 (b), 5 (c) and 40 mg/dm³ (d).

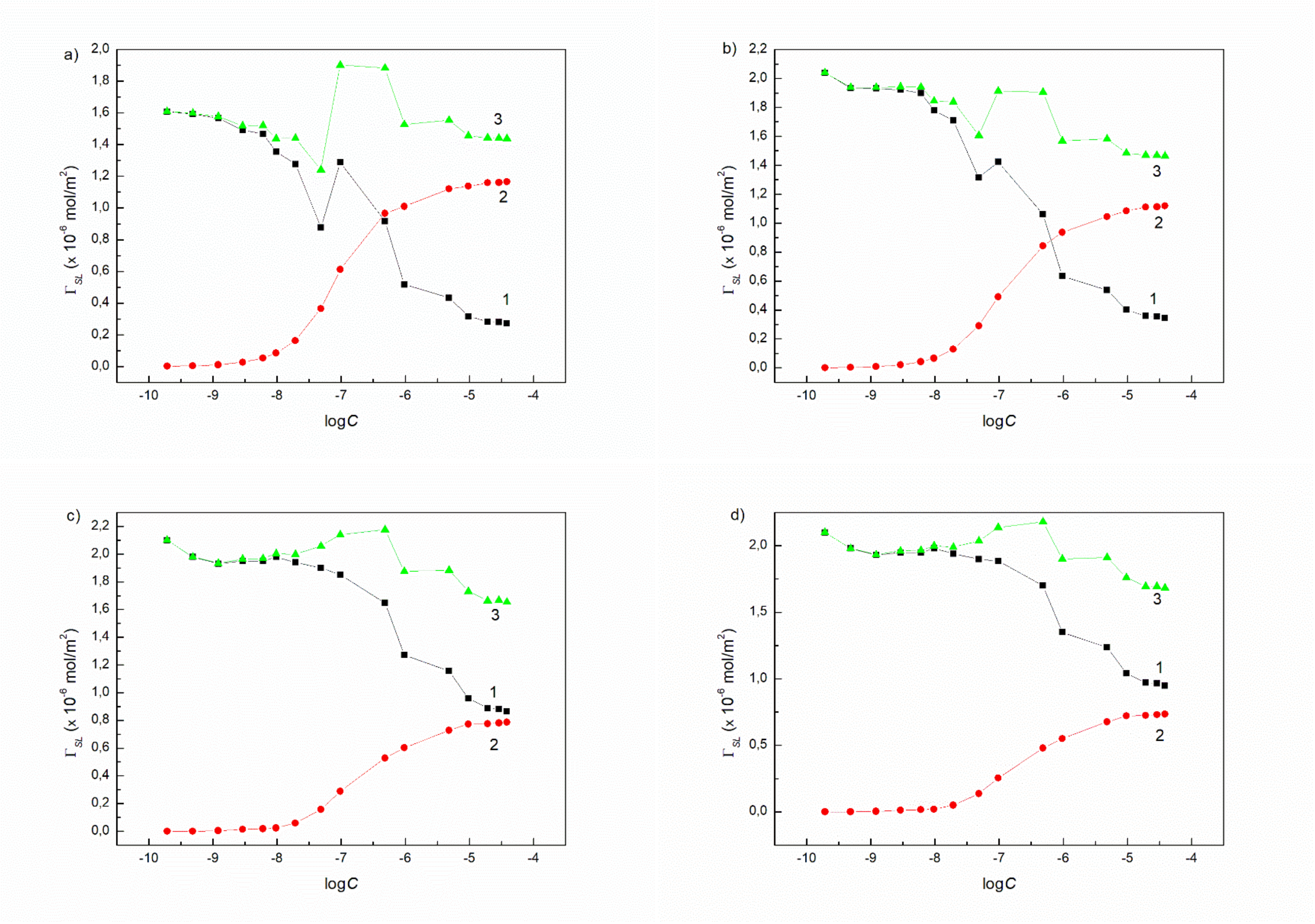

Figure S31. A plot of the Gibbs surface excess concentration at the PTFE-water interface (Γ_{SL}) for TX165 (curve 1), SF (2) and their sum (curve 3) vs. the logarithm of surfactant concentration (C) at the constant TX165 concentration equal to 5 × 10^{-7} (a), 1 × 10^{-6} (b), 2 ×10^{-4} (c) and 1 × 10^{-3} mole/dm^3 (d).

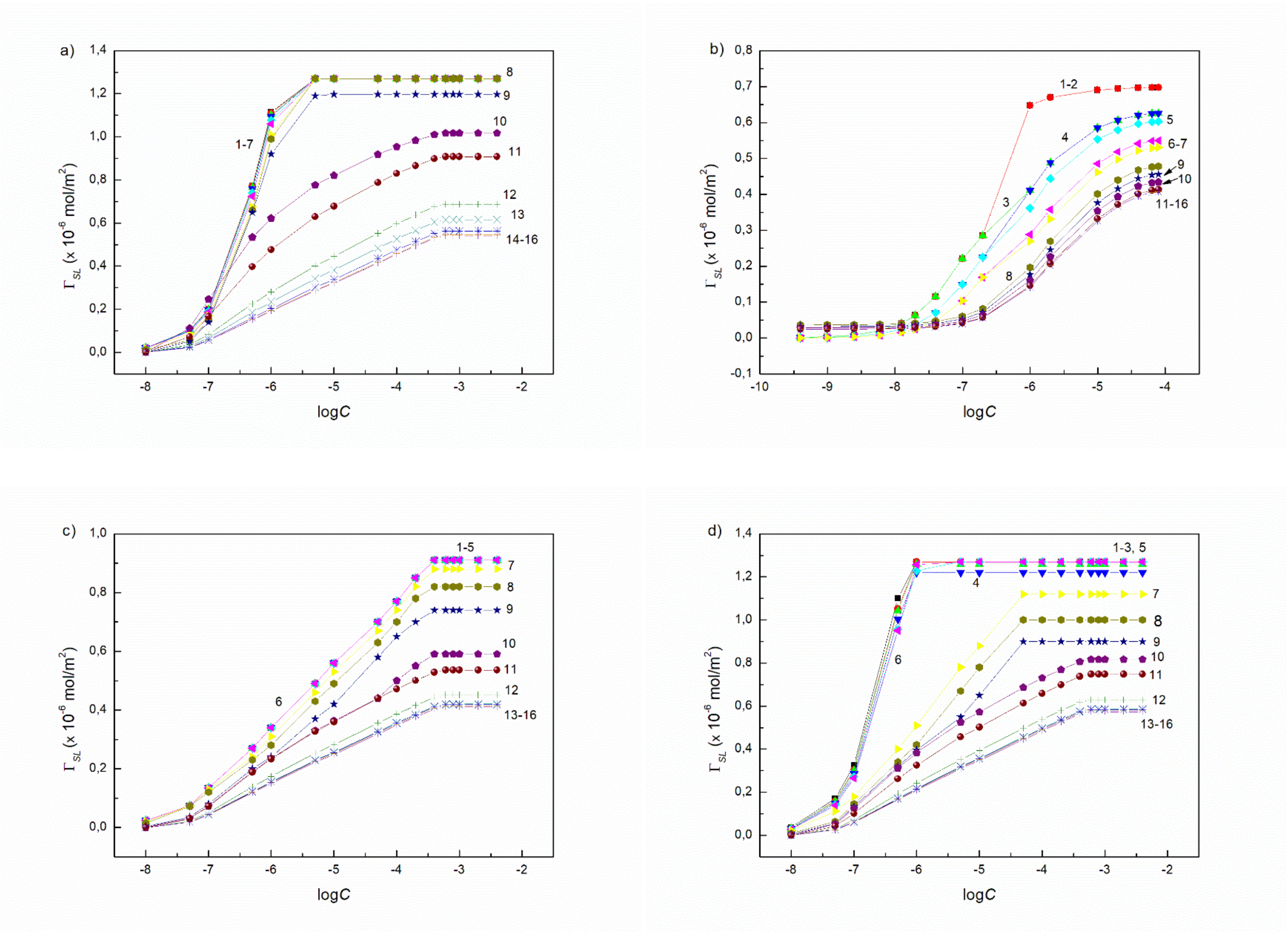

Figure S32. A plot of the Gibbs surface excess concentration at the PMMA-water interface (Γ_{SL}) vs. the logarithm of surfactant concentration (C) for TX165 + RL (a, b) and TX165 +SF (c, d) at the constant TX165 concentration (b, d) and RL (a) as well as SF (c).

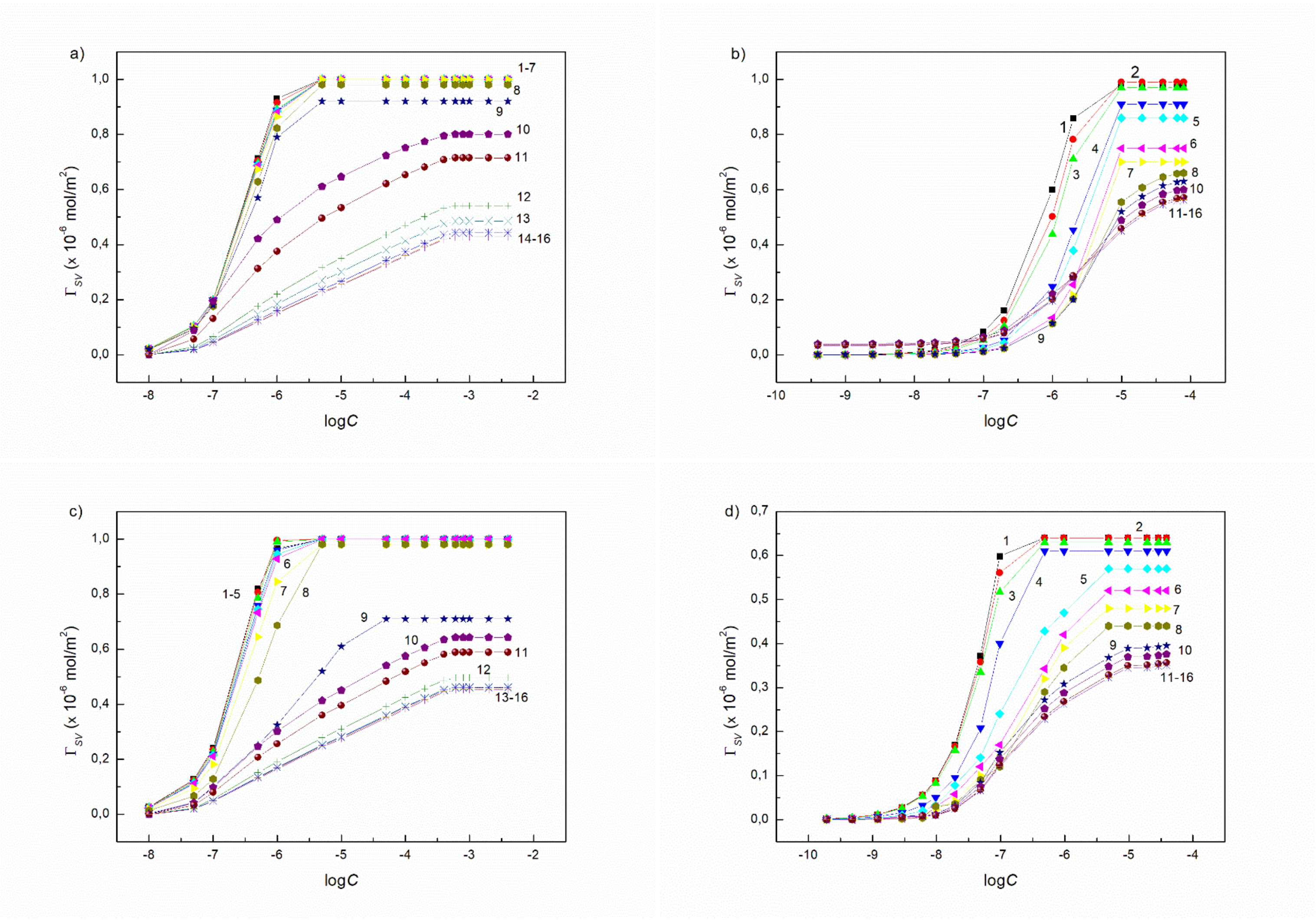

Figure S33. A plot of the Gibbs surface excess concentration at the PMMA-air interface (Γ_{SV}) vs. the logarithm of surfactant concentration (C) for TX165 + RL (a, b) and TX165 +SF (c, d) at the constant TX165 concentration (b, d) and RL (a) as well as SF (c).

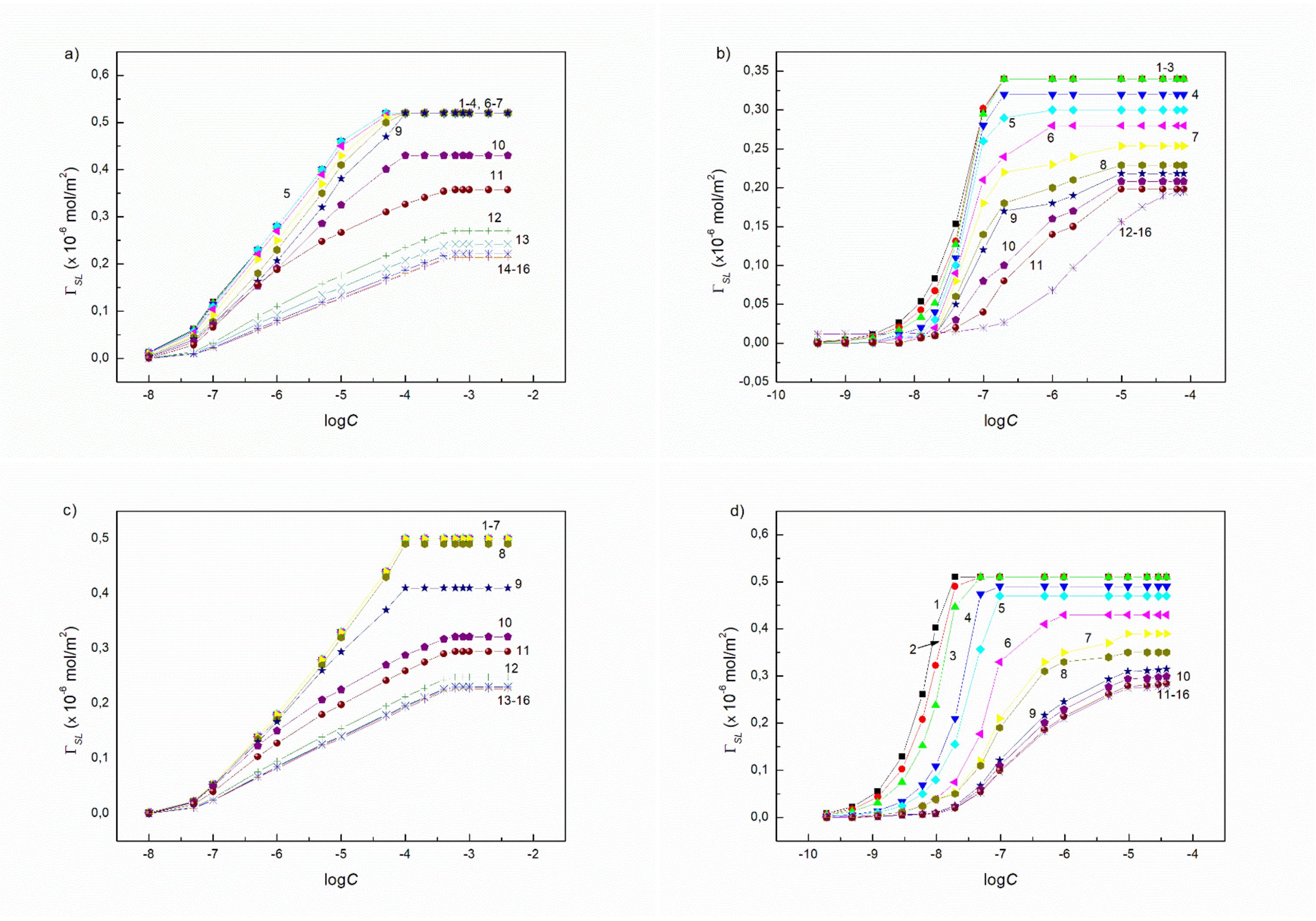

Figure S34. A plot of the Gibbs surface excess concentration at the quartz-water interface (Γ_{SL}) vs. the logarithm of surfactant concentration (C) for TX165 + RL (a, b) and TX165 +SF (c, d) at the constant TX165 concentration (b, d) and RL (a) as well as SF (c).

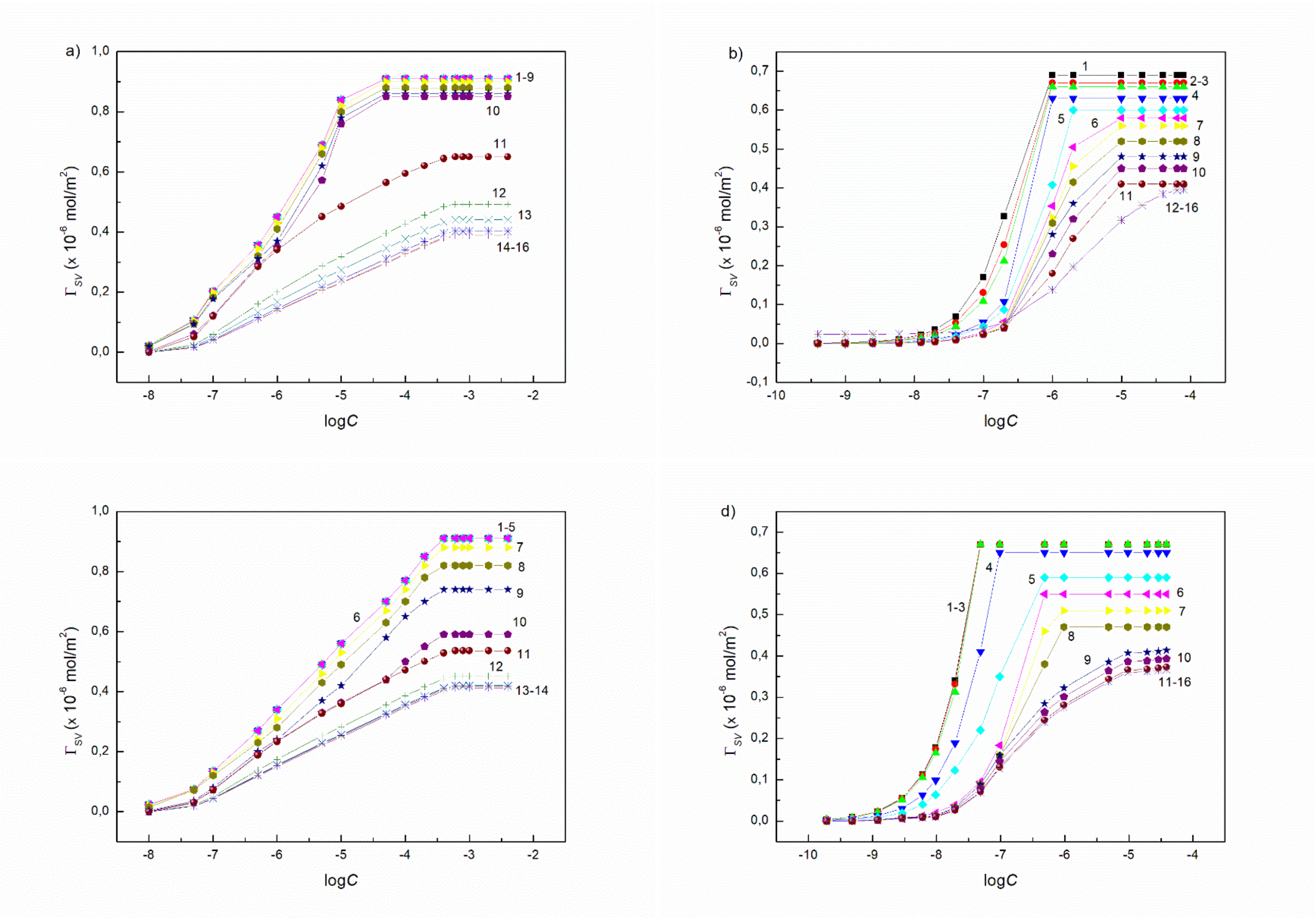

Figure S35. A plot of the Gibbs surface excess concentration at the quartz-air interface (Γ_{SV}) vs. the logarithm of surfactant concentration (C) for TX165 + RL (a, b) and TX165 +SF (c, d) at the constant TX165 concentration (b, d) and RL (a) as well as SF (c).

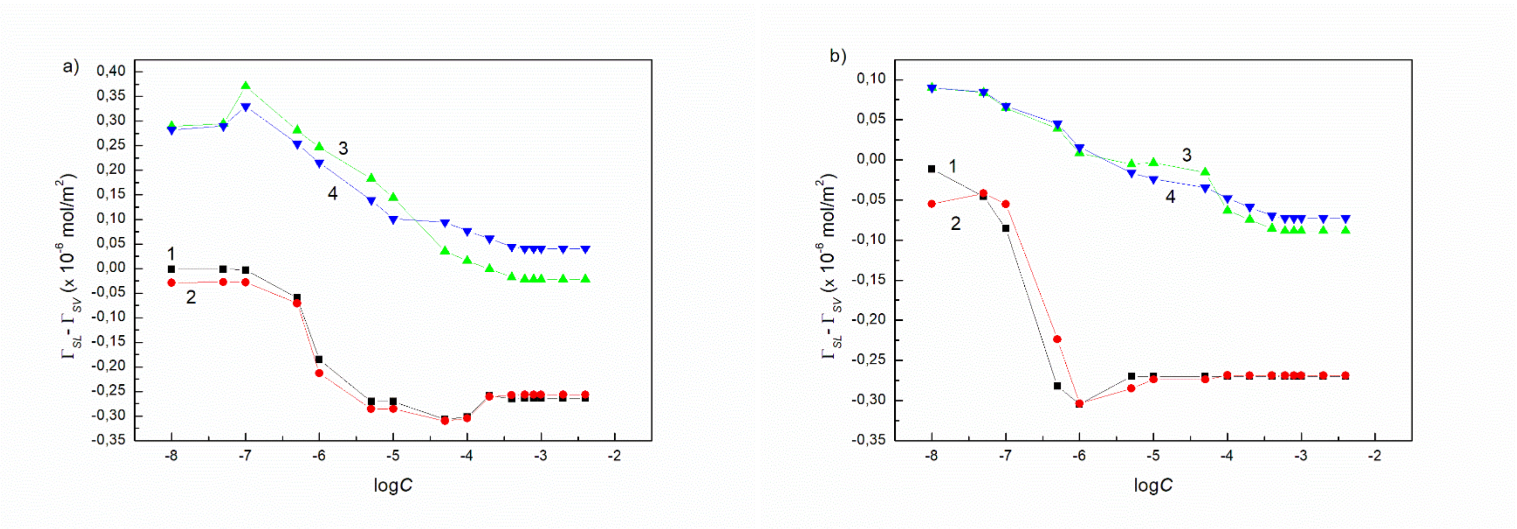

Figure S36. The difference between the Gibbs surface excess concentration at the PMMA-water and PMMA-air interface vs. the logarithm of surfactant concentration (C) for TX165 + RL (a) and TX165 +SF (b). Curves 1 – 4 correspond to the constant biosurfactant concentration equal to 0.0002, 0.00625, 5 and 40 mg/dm^3.

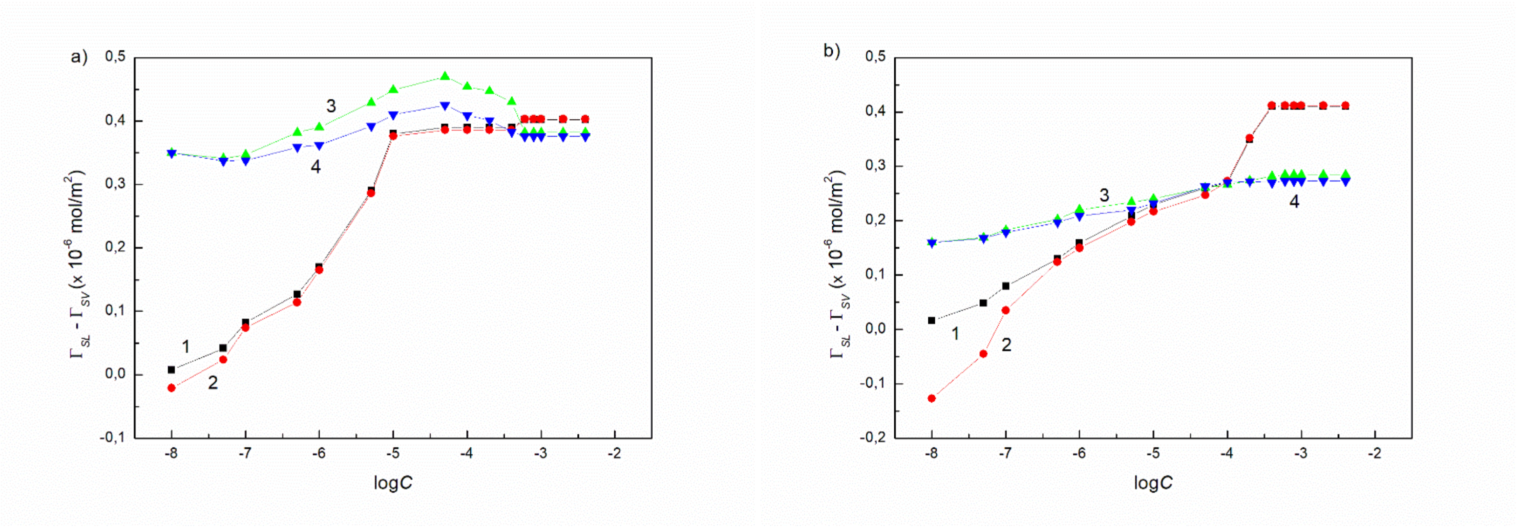

Figure S37. A plot of the difference between the Gibbs surface excess concentration at the quartz-water and quartz-air interface vs. the logarithm of surfactant concentration (C) for TX165 + RL (a) and TX165 +SF (b). Curves 1 – 4 correspond to the constant biosurfactant concentration equal to 0.0002, 0.00625, 5 and 40 mg/dm^3.

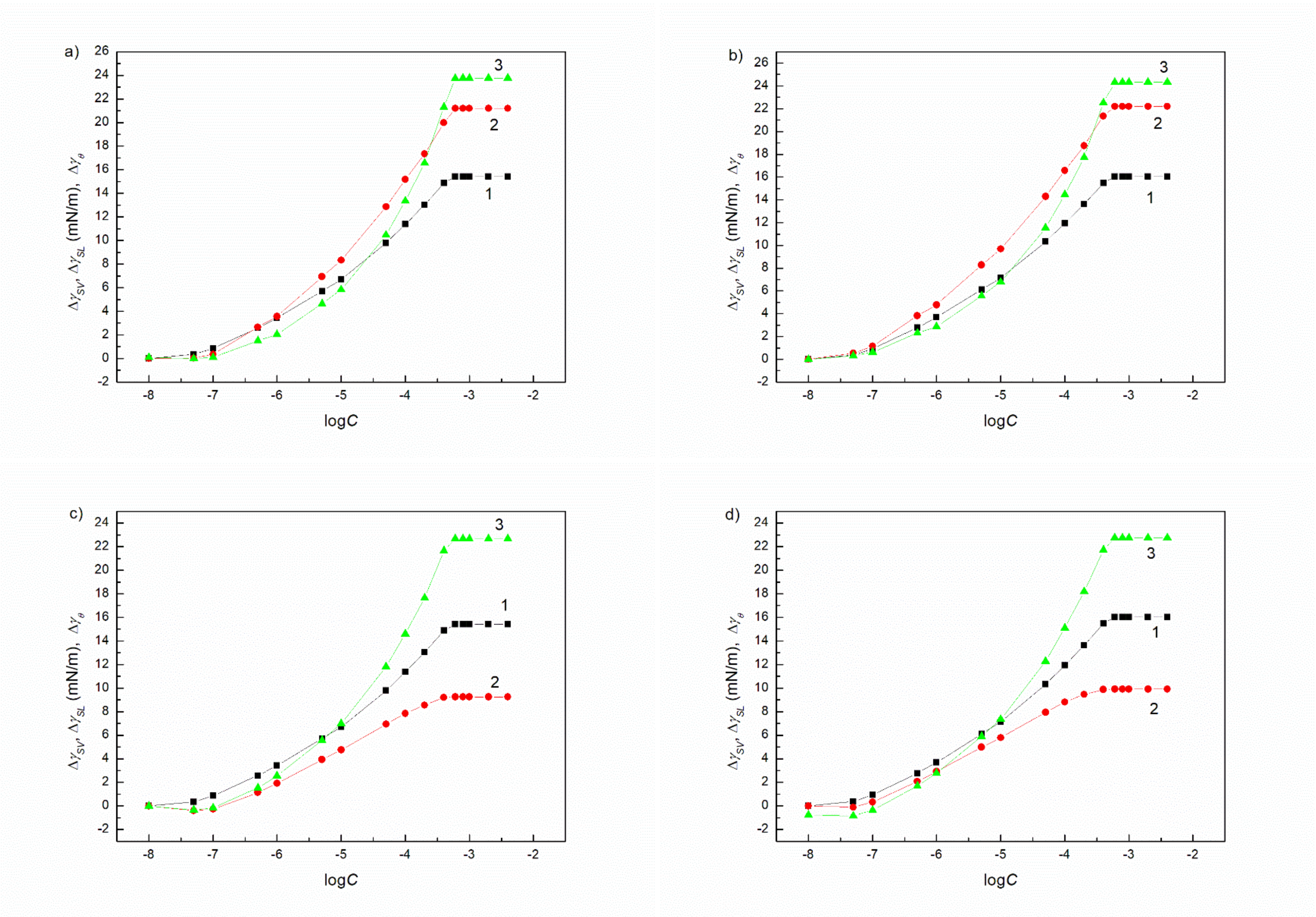

Figure S38. A plot of the difference between the values of the PMMA(quartz)-air interface tension ($\Delta\gamma_{SV}$) at the TX165 + biosurfactant mixture concentration equal to zero and equal to a given value (curve 1) and between the PMMA(quartz)-water (curve 2) and PMMA(quartz)-solution (curve 2) ($\Delta\gamma_{SL}$) as well as the difference between the values of contact angle of the water and solution on the PMMA (a, b) and quartz (c, d) ($\Delta\gamma_{\theta}$) (curve 3) at the constant RL and SF concentration equal to 0.00625 mg/dm3 vs. the logarithm of surfactant concentration (C).
